# Complications associated using the reamer–irrigator –aspirator (RIA) system: a systematic review and meta-analysis

**DOI:** 10.1007/s00402-022-04621-z

**Published:** 2022-09-17

**Authors:** Markus Laubach, Lucas P. Weimer, Felix M. Bläsius, Frank Hildebrand, Philipp Kobbe, Dietmar W. Hutmacher

**Affiliations:** 1 Australian Research Council (ARC) Training Centre for Multiscale 3D Imaging, Modelling, and Manufacturing (M3D Innovation), Queensland University of Technology, Brisbane, QLD 4000 Australia; 2 Department of Orthopaedics, Trauma and Reconstructive Surgery, RWTH Aachen University Hospital, Pauwelsstraße 30, 52074 Aachen, Germany; 3 Faculty of Health, Medicine and Life Sciences, Maastricht University, Universiteitssingel 40, 6229 ER Maastricht, The Netherlands; 4 Australian Research Council (ARC) Training Centre for Cell and Tissue Engineering Technologies, Queensland University of Technology (QUT), Brisbane, QLD 4000 Australia; 5 Max Planck Queensland Center for the Materials Science of Extracellular Matrices, Queensland University of Technology, Brisbane, QLD 4000 Australia

**Keywords:** Reamer–Irrigator–Aspirator, Complications, Reaming, Blood loss, Review

## Abstract

**Introduction:**

Complications associated with the application of the Reamer–irrigator–Aspirator (RIA) system are described in the literature. However, to date a systematic review and meta-analysis to assess prevalence of complications associated with the use of the RIA system have not been conducted.

**Materials and methods:**

The review is registered with PROSPERO (CRD42021269982). MEDLINE, the Web of Science Core Collection, and Embase were searched from the inception to 10 August 2021. The primary objective was to assess complications and blood loss associated with the use of the RIA system.

**Results:**

Forty-seven studies involving 1834 procedures performed with the RIA system were finally included. A total of 105 complications were reported, with a pooled estimated overall prevalence of 1.7% with a 95% confidence interval (CI) of 0.40 to 3.60, with cortex perforation being the largest reported complication with a total of 34 incidences. A significant subgroup difference was observed (*p* = 0.02). In subgroup 1 (bone graft harvesting), complication prevalence was 1.4% (95% CI 0.2–3.4); in subgroup 2 (clearance intramedullary canal) it was 0.7% (95% CI 0.00–6.30) and in subgroup 3 (reaming with RIA system prior to nail fixation) 11.9% (95% CI 1.80–26.40). No statistically significant difference for tibia and femur as RIA system application site was observed (CI 0.69–4.19). In studies reporting blood loss, a mean volume of 803.29 ml, a mean drop of hemoglobin of 3.74 g/dl and a necessity of blood transfusion in 9.72% of the patients were observed.

**Conclusions:**

The systematic review and meta-analysis demonstrate a low overall prevalence rate of complications associated with the RIA system. However, especially the risk of cortical perforation and the frequently reported relevant intraoperative blood loss are complications that should be anticipated in perioperative management and ultimately considered when using the RIA system.

**Supplementary Information:**

The online version contains supplementary material available at 10.1007/s00402-022-04621-z.

## Introduction

The Reamer–Irrigator–Aspirator (RIA) system (Synthes, Inc., West Chester, PA), which provides continuous irrigation and suction during the reaming of long bones, was first developed to reduce the incidence of fat embolism (FE) and thermal necrosis [1, 2]. Although often times neglected in the current literature, it were Dankwardt–Lillieström and colleagues, who more than 50 years ago originated the suction irrigation reaming method and demonstrated in a preclinical rabbit model that clearance of the medullary canal of long bones prior to reaming or nail fixation might result in improved bone healing and less FE by reducing the expulsion of bone marrow into the blood vessels of the cortical bone [3–6]. Rooted in their concept and subsequent (technical) device advancements, the RIA system was attributed a potentially important role in the prevention of FE and a favorable local effect on the microstructure of the bone and, thus, on fracture healing [1, 6–9]. Therefore, clearing the medullary canal of bone marrow-rich reaming debris prior to intramedullary nailing was the primary indication for the RIA system, for which it was approved by the FDA in 2000 [10].

The osteogenic potential of reaming debris, particularly originating from the endocortex, was first described more than 30 years ago; however, its extraction from the reamer head was tedious [11, 12]. Indeed, the literature indicates no differences in union rate comparing autologous bone graft (ABG) harvested from the iliac crest (IC) as the gold standard compared to RIA graft [13, 14]. With the RIA system, clinicians soon recognized the promising capacity to harvest large quantities of endosteal ABG from the intramedullary cavity of lower leg long bones [15, 16]. Therefore, due to the ability to harvest large amounts of ABG with a filter system, the RIA system was repurposed as an ABG collector. In addition, less donor site pain, fewer infections, and a lower rate of adverse events have been observed with the use of the RIA system, and the cost of its use may be offset by the time saved in the operating room, especially when compared to harvesting posterior ICBG [13, 14]. Thus, the RIA system was approved by the FDA in 2005 for obtaining ABG associated with a large harvesting capacity [10].

However, application of the RIA system can also cause serious complications. The RIA system is considered an one-time aggressive reamer with continuous irrigation at the reamer head that can cause eccentric reaming and blood loss, causing iatrogenic fractures and cardiopulmonary disturbances, respectively [17, 18]. However, precisely these features of powerful evacuation on medullary canal content furthered the application of the RIA device as a multipurpose tool to lower the risk of dissemination into the soft tissues and systemic circulation as well as clearing the femoral/tibial canal of cement debris. Indeed, preclinical large animal studies showed that application of the RIA system was associated with a reduced amount of FE [19, 20] and lower systemic effects compared to conventional reaming [21, 22]. Based on these findings, the RIA system has been applied to reduce intravasation of intramedullary content, such as malignancies [23], infections [24], and cement removal [25]. However, systematic reporting of the associated complications is essential; yet, surprisingly, no systematic review of the complications associated with the use of the RIA system has been conducted.

The aim of this systematic review and meta-analysis was to assess the prevalence of intra- and postoperative complications in patients treated with the RIA system.

## Materials and methods

This systematic review and meta-analysis were conducted in accordance with the Preferred Reporting Items for Systematic Reviews and Meta-Analyses (PRISMA) 2020 statement (PRISMA 2020 checklist available in Supplementary Material 1) [26] in conjunction with the associated literature search extension PRISMA-S [27]. This review has been registered with PROSPERO: CRD42021269982. Search strategy development and documentation were improved by close collaboration with a librarian at the Queensland University of Technology.

### Eligibility criteria

A framework for the identification of studies eligible for inclusion was applied as per the components of population, exposure of interest, and outcome in accordance with Moola et al. 2020 [28]. Accordingly, studies were included if they were conducted in patients (no age constraints) who were treated with the RIA system irrespective of specific clinical/surgical indications (population). The original RIA system (original RIA/RIA 1) has recently been updated, and a new version—termed RIA 2 system—is now available [29]. Therefore, all clinical studies reporting complications associated with the use of both systems were included (exposure of interest). Eligible studies provided data on any intra- or postoperative complications or sequalae during follow-up related to the RIA system application (outcome). The exclusion criteria entailed studies that did not use the RIA system or did not assess complications related to the RIA system; studies published in languages other than English, Dutch or German; case reports with less than two cases; review articles; in vitro studies and conference abstracts.

### Search strategy

MEDLINE (via PubMed), Web of Science Core Collection (Clarivate Analytics), and Embase (via Elsevier) were the databases used for the computerized search strategy. The systematic search was performed on 10 August 2021 (PRISMA-S checklist available in Supplementary Material 2). The strategy was modified and adjusted for each database searched, with no restrictions on language or time period. Detailed search strategies used are available in Supplementary Material 3. Following the literature search, all identified research reports were collated and uploaded to EndNote 20.0.1 (Clarivate Analytics, PA, USA) and duplicates removed. Subsequently, titles and abstracts were screened for inclusion by two independent reviewers (ML and LPW). Full texts were obtained for the eligible studies and screened for inclusion. Reference lists of the included studies were manually screened to identify additional studies for inclusion. All study details were imported into the Joanna Briggs Institute System for the Unified Management, Assessment and Review of Information (JBI SUMARI, Adelaide, Australia), and reasons for exclusion of full-text studies were recorded.

### Data collection

Relevant data was retrieved manually and included the following: study characteristics (authors, year of publication, level of evidence), study design (prospective/retrospective), RIA system procedure details (total number per study, indication), surgical approach including anatomical site of reamed bone (femur/tibia) and bone access (antegrade/retrograde). In addition, the outcome measures were documented, including intraoperative cortex perforations requiring internal fixation or not requiring internal fixation, low energy bone fracture during follow-up, RIA device failure including reamer assembly disengagement, broken tip of the RIA drive shaft or metallic debris, cardiopulmonary complications and systemic infection, nerve injuries, hematoma/hemarthrosis and wound or local infection. In case of missing data, the respective authors were contacted.

### Data synthesis and analysis

In the first step, the extracted data on the study characteristics and outcome measures are presented descriptively. As per the clinical indication to apply the RIA system three subgroups were formed and complication prevalence assessed accordingly. The first subgroup included all studies that reported on the application for ABG harvesting, the second subgroup on clearance of the intramedullary canal (treatment of osseous infections or bone malignancy and removing intramedullary cement) and the third subgroup for reamed nail fixation. Further, the number of total complications per surgical site (femur/tibia) were compared. If ten or more complications per outcome measure or surgical site (tibia/femur) were present, a meta-analysis was performed using a random effects model.

Statistical analyses of the data required for the meta-analyses, as well as creation of the forest plots, were performed using R programming software (version 4.0.2; R Foundation for Statistical Computing, Vienna, Austria) with RStudio version 1.3.1073 (RStudio Inc., Boston, MA). The R software packages used to conduct the meta-analyses of proportions using a random effects model were ‘metafor’ [30] and ‘meta’ [31, 32]. The forest plot showing the odds ratios (OR) of the application sites of femur and tibia was created with the R software package ‘metafor’ [30]. The preferred heterogeneity variance estimator of restricted maximum likelihood (REML) [33] was applied. To transform prevalence estimates for proportional meta-analysis, the recommended method of the Freeman–Tukey double arcsine transformation was used [34, 35]. The meta-analyses are reported grouped by a random effects model [36] and presented with 95% confidence intervals (CI) [35]. Values of *p* < 0.05 were considered statistically significant. Heterogeneity between studies was assessed visually by means of forest plots and quantitatively measured by the index of heterogeneity squared (*I*^2^) statistics with 95% CIs and are considered low, moderate and high where *I*^2^ values are below 25%, between 25 and 75% and above 75%, respectively [37].

### Risk of bias assessment

Two authors (ML and LPW) independently assigned the level of evidence according to the recommendations of the Centre for Evidence-Based Medicine (Oxford, United Kingdom) [38] and assessed the methodologic quality and risk of bias using the JBI critical appraisal tools applicable for the different study types [28, 39, 40]. Any disagreements were resolved through discussion or, if necessary, the senior author's (DWH) judgment was decisive. The appraisal tools are listed in Supplementary Material 4. Funnel plots were used to illustrate the risk of publication bias. The funnel plot for the analyses using proportional data was created with the R package ‘berryFunctions’, and for the subgroup analysis of comparing the tibia and femur as RIA system application site the funnel plot was created with the R package ‘metafor’.

## Results

**Fig. 1:**
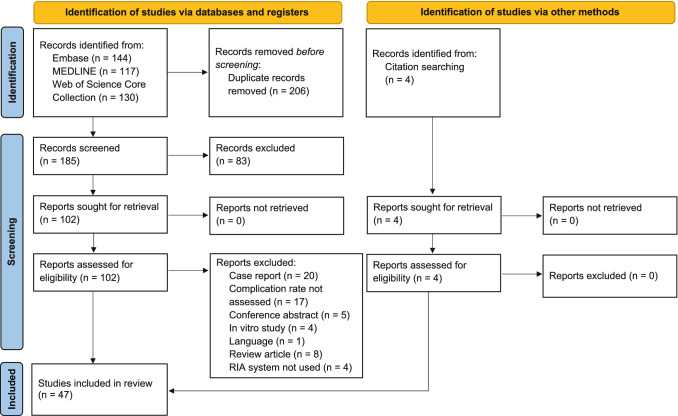
PRISMA 2020 flowchart [26]

The database search identified 185 publications after duplicates were removed. A total of 83 articles did not match the inclusion criteria and were excluded after title and abstract screening. Four additional studies were identified via citation searching, resulting in 106 articles eligible for full-text assessment. Full-text assessment resulted in the exclusion of 59 studies, and reasons for exclusion of these articles are listed in Supplementary Material 5. Thus, 47 studies were included in the qualitative and quantitative synthesis. Figure 1 depicts the PRISMA 2020 flowchart with the study selection procedure.

### Study characteristics and findings

**Fig. 2:**
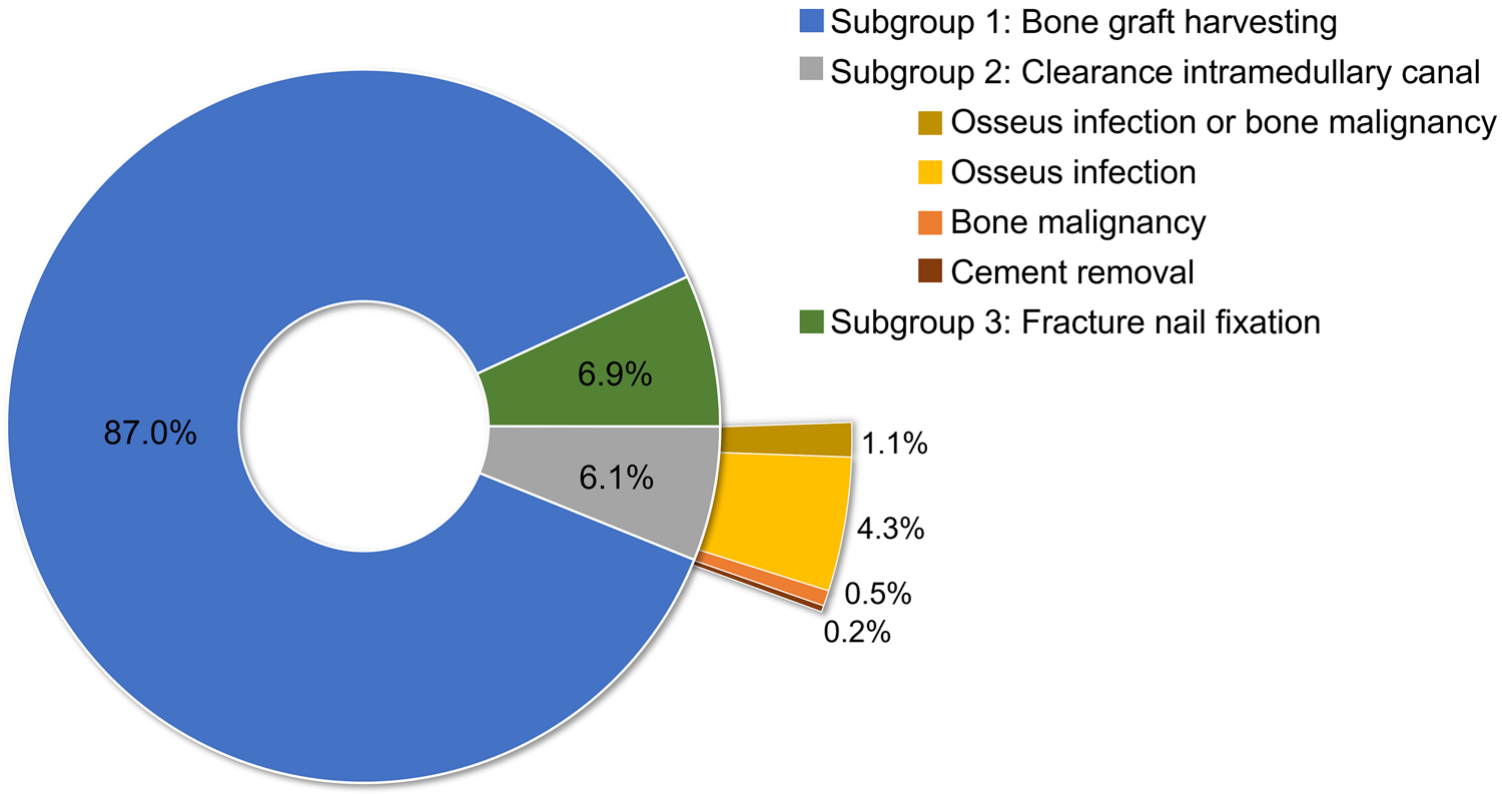
Bar of pie chart showing the subgroup distribution of the RIA system indication

Characteristics of the included studies are shown in Table 1. The total number of included RIA system procedures was 1834, of which 1677 (91.4%) were performed on the surgical site of femur and 157 (8.6%) on the tibia. In 87.0% (1,596/1834) of the cases, the RIA device was applied for ABG harvesting (subgroup 1); 6.1% (112/1834) for clearance of the intramedullary canal, which included osseous infection, bone malignancy and cement removal (subgroup 2) and 6.9% (126/1834) for intramedullary reaming prior to nail fixation (subgroup 3) (Fig. 2). In total, 105 complications were reported in 1834 procedures performed with the RIA device. Intraoperative cortex perforations account for the most complications (34/1834), followed by cardiopulmonary complications/systemic infection (29/1834). All findings are listed in Table 2.

**Table 1:** Study characteristics

Authors/Year of publication	Study design	Level of evidence	Prospective/retrospective	Total number of RIA system procedures	RIA system indication (number of procedures, if applicable for multiple groups)	Anatomical site (if applicable number of procedures)	Surgical access (number of procedures, if applicable for multiple groups)
Barlow and Kuhn (2014) [55]	Case series	4	Retrospective	3	ABG harvesting	Femur	Retrograde
Belthur et al. (2008) [41]	Cohort study	3	Retrospective	41	ABG harvesting	Femur (37) Tibia (4)	Antegrade
Calori et al. (2014) [56]	Cohort study	3	Retrospective	35	ABG harvesting	Femur	Antegrade
Cipriano et al. (2012) [23]	Case series	4	Retrospective	21	Treatment of osseous infections or bone malignancy	Femur	Antegrade
Conway et al. (2014) [77]	Case series	4	Retrospective	15	ABG harvesting	Femur	Antegrade (9) Retrograde (6)
Davis et al. (2015) [78]	Cohort study	3	Retrospective	94	ABG harvesting	Femur	Antegrade (62) Retrograde (32)
Dawson et al. (2014) [14]	Randomized controlled trial	1	Prospective	56	ABG harvesting	Femur	Antegrade
Donders et al. (2016) [18]	Case series	4	Retrospective	2	ABG harvesting	Femur	Antegrade (1) Retrograde (1)
Eisenstein et al. (2016) [79]	Case series	4	Retrospective	6	ABG harvesting	Femur	Retrograde
Grote et al. (2015) [57]	Case series	4	Prospective	5	ABG harvesting	Femur	Antegrade
Hall et al. (2017) [69]	Randomized controlled trial	1	Prospective	11	Fracture stabilization	Femur	Antegrade
Han et al. (2015) [42]	Case series	4	Retrospective	57	ABG harvesting	Femur: (43) Tibia (14)	Antegrade
Haubruck et al. (2018) [43]	Case series	4	Retrospective	341	ABG harvesting	Femur (336) Tibia (5)	Antegrade (Femur: 336; Tibia: 1) Retrograde (Tibia: 4)
Herscovici and Scaduto (2012) [80]	Case series	4	Retrospective	30	ABG harvesting	Tibia	Antegrade (3) Retrograde (27)
Jakma et al. (2014) [81]	Case series	4	Retrospective	32	ABG harvesting	Femur	Antegrade (31) Retrograde (1)
Kanakaris et al. (2011) [67]	Case series	4	Prospective	42	ABG harvesting (18)	Femur	Antegrade
					Fracture stabilization (7)		
					Treatment of osseous infections (8) or bone malignancy (9)		
Kanakaris et al. (2014) [44]	Case series	4	Prospective	24	Treatment of osseous infections	Femur (14) Tibia (10)	Antegrade
Krappinger et al. (2015) [82]	Case series	4	Retrospective	4	ABG harvesting	Femur	Antegrade
Kusnezov et al. (2017) [83]	Case series	4	Retrospective	15	ABG harvesting	Femur	Antegrade
Le Baron et al. (2019) [45]	Cohort study	3	Prospective	30	ABG harvesting	Femur (16) Tibia (14)	Antegrade
Lehman et al. (2012) [84]	Case series	5	Retrospective	3	ABG harvesting	Tibia	Retrograde
Lowe et al. (2010) [46]	Case series	4	Retrospective	97	ABG harvesting	Femur (93) Tibia (4)	Antegrade
Lowe et al. (2011) [25]	Case series	5	Retrospective	3	Removing intramedullary cement	Femur (1) Tibia (2)	Antegrade
Mansour and Conway (2015) [85]	Case series	5	Retrospective	2	ABG harvesting	Femur	Retrograde
Marchand et al. (2017) [47]	Cohort study	3	Retrospective	62	ABG harvesting	Femur (49) Tibia (13)	Antegrade for harvesting in tibia; Retrograde and antegrade in femur (depending on non-union location)
Marko et al. (2016) [58]	Case series	5	Retrospective	2	ABG harvesting	Femur	Antegrade
Martella et al. (2021) [48]	Cohort study	3	Retrospective	65	ABG harvesting	Femur (60) Tibia (5)	Antegrade
McCall et al. (2010) [49]	Case series	4	Prospective	24	ABG harvesting	Femur (22) Tibia (2)	Antegrade
Metsemakers et al. (2019) [86]	Case series	4	Retrospective	72	ABG harvesting	Femur	Antegrade
Moghaddam et al. (2017) [87]	Case series	4	Retrospective	76	ABG harvesting	Femur	Antegrade
Moghaddam et al. (2015) [74]	Cohort study	2	Prospective	48	ABG harvesting	Femur	Antegrade
Naruka et al. (2019) [88]	Case series	4	Retrospective	3	ABG harvesting	Femur	Antegrade
Newman et al. (2008) [89]	Case series	4	Retrospective	10	ABG harvesting	Femur	Antegrade
Niikura et al. (2021) [50]	Case series	4	Retrospective	42	ABG harvesting (32)	Femur (40) Tibia (2)	Antegrade
					Fracture stabilization (1)		
					Treatment of osseous infections (9)		
Nodzo et al. (2014) [90]	Cohort study	3	Retrospective	29	ABG harvesting	Femur	Antegrade
O’Callaghan et al. (2019) [59]	Case series	5	Retrospective	2	ABG harvesting	Femur	Antegrade
Onsea et al. (2021) [51]	Cohort study	3	Retrospective	24	Treatment of osseous infections	Femur (12) Tibia (12)	Antegrade
Quintero et al. (2010) [60]	Case series	5	Retrospective	20	ABG harvesting	Femur	Antegrade (19) Retrograde (1)
Qvick et al. (2013) [52]	Case series	4	Retrospective	204	ABG harvesting (201)	Femur (179) Tibia (25)	Antegrade (Femur: 175; Tibia: 7) Retrograde (Femur: 4; Tibia: 18)
					Treatment of osseous infections (3)		
Rauck et al. (2016) [91]	Case series	5	Retrospective	2	ABG harvesting	Femur	Antegrade
Seagrave et al. (2014) [92]	Case series	4	Retrospective	3	ABG harvesting	Femur	Retrograde
Stafford and Norris (2010) [93]	Case series	4	Retrospective	27	ABG harvesting	Femur	Antegrade
Streubel et al. (2010) [70]	Case series	4	Retrospective	97	Fracture stabilization	Femur	Antegrade (30) Retrograde: (53); Greater trochanter (11) Lateral femur (6)
Volgas et al. (2010) [75]	Randomized controlled trial	1	Prospective	10	Fracture stabilization	Femur	Antegrade
Walker et al. (2019) [94]	Case series	4	Retrospective	8	ABG harvesting	Femur	Antegrade
Waterman et al. (2017) [53]	Case series	4	Prospective	24	ABG harvesting	Femur (20) Tibia (4)	Antegrade
Zalavras et al. (2007) [54]	Case series	4	Retrospective	11	Treatment of osseous infections	Femur (3) Tibia (8)	Antegrade

**Table 2:** Incidence of donor site morbidity following use of the RIA system

Authors/Year of publication	Intra-operative cortex perforations		Low energy bone fracture during follow-up	RIA device failure (reamer assembly disengagement, broken tip of the RIA drive shaft or metallic debris)	Cardio-pulmonary complications/systemic infection	Nerve injuries	Hematoma/hemarthrosis	Wound or local infection	Total complications
	No internal fixation performed	Internal fixation was performed							
Barlow and Kuhn (2014) [55]	0/3	0/3	0/3	0/3	0/3	0/3	0/3	0/3	0/3
Belthur et al. (2008) [41]	1/41	1/41	0/41	2/41	0/41	0/41	0/41	0/41	4/41
Calori et al. (2014) [56]	0/35	0/35	0/35	2/35	0/35	0/35	0/35	0/35	2/35
Cipriano et al. (2012) [23]	0/21	0/21	0/21	2/21	0/21	0/21	0/21	0/21	2/21
Conway et al. (2014) [77]	0/15	0/15	0/15	0/15	0/15	0/15	0/15	0/15	0/15
Davis et al. (2015) [78]	0/94	4/94	0/94	0/94	0/94	0/94	0/94	0/94	4/94
Dawson et al. (2014) [14]	0/56	0/56	1/56	0/56	0/56	0/56	0/56	0/56	1/56
Donders et al. (2016) [18]	0/2	0/2	0/2	0/2	2/2	0/2	0/2	0/2	2/2
Eisenstein et al. (2016) [79]	0/6	0/6	0/6	1/6	0/6	0/6	0/6	0/6	1/6
Grote et al. (2015) [57]	0/5	0/5	0/5	0/5	0/5	0/5	0/5	0/5	0/5
Hall et al. (2017) [69]	0/11	1/11	0/11	0/11	1/11	0/11	0/11	0/11	2/11
Han et al. (2015) [42]	0/57	1/57	0/57	0/57	0/57	0/57	0/57	2/57	3/57
Haubruck et al. (2018) [43]	0/341	1/341	2/341	1/341	2/341	0/341	1/341	1/341	8/341
Herscovici and Scaduto (2012) [80]	1/30	0/30	0/30	0/30	0/30	0/30	0/30	0/30	1/30
Jakma et al. (2014) [81]	6/32	1/32	0/32	0/32	3/32	0/32	0/32	0/32	10/32
Kanakaris et al. (2011) [67]	0/42	0/42	0/42	1/42	0/42	0/42	2/42	0/42	3/42
Kanakaris et al. (2014) [44]	1/24	0/24	0/24	0/24	0/24	0/24	0/24	0/24	1/24
Krappinger et al. (2015) [82]	0/4	0/4	0/4	0/4	0/4	0/4	0/4	0/4	0/4
Kusnezov et al. (2017) [83]	0/15	0/15	1/15	0/15	0/15	0/15	0/15	0/15	1/15
Le Baron et al. (2019) [45]	0/30	0/30	0/30	0/30	0/30	0/30	1/30	0/30	1/30
Lehman et al. (2012) [84]	0/3	0/3	0/3	0/3	0/3	0/3	0/3	0/3	0/3
Lowe et al. (2010) [46]	1/97	1/97	4/97	0/97	0/97	0/97	0/97	0/97	6/97
Lowe et al. (2011) [25]	0/3	0/3	0/3	0/3	0/3	0/3	0/3	0/3	0/3
Mansour and Conway (2015) [85]	0/2	0/2	0/2	0/2	0/2	0/2	0/2	0/2	0/2
Marchand et al. (2017) [47]	0/62	0/62	0/62	0/62	0/62	0/62	0/62	1/62	1/62
Marko et al. (2016) [58]	0/2	0/2	0/2	0/2	1/2	0/2	0/2	0/2	1/2
Martella et al. (2021) [48]	0/65	0/65	0/65	0/65	2/65	0/65	0/65	0/65	2/65
McCall et al. (2010) [49]	0/24	0/24	0/24	0/24	0/24	0/24	0/24	0/24	0/24
Metsemakers et al. (2019) [86]	3/72	2/72	0/72	0/72	0/72	0/72	0/72	0/72	5/72
Moghaddam et al. (2017) [87]	0/76	0/76	0/76	0/76	0/76	0/76	0/76	0/76	0/76
Moghaddam et al. (2015) [74]	0/48	0/48	0/48	0/48	0/48	0/48	0/48	0/48	0/48
Naruka et al. (2019) [88]	0/3	0/3	0/3	0/3	0/3	0/3	0/3	0/3	0/3
Newman et al. (2008) [89]	0/10	0/10	0/10	0/10	0/10	0/10	0/10	0/10	0/10
Niikura et al. (2021) [50]	5/42	0/42	1/42	0/42	0/42	0/42	0/42	0/42	6/42
Nodzo et al. (2014) [90]	0/29	0/29	0/29	0/29	0/29	0/29	0/29	2/29	2/29
O’Callaghan et al. (2019) [59]	0/2	0/2	0/2	0/2	0/2	0/2	0/2	0/2	0/2
Onsea et al. (2021) [51]	0/24	0/24	0/24	0/24	0/24	0/24	0/24	0/24	0/24
Quintero et al. (2010) [60]	2/20	0/20	0/20	0/20	1/20	0/20	0/20	0/20	3/20
Qvick et al. (2013) [52]	1/204	1/204	2/204	0/204	0/204	0/204	0/204	0/204	4/204
Rauck et al. (2016) [91]	0/2	0/2	0/2	0/2	0/2	0/2	0/2	0/2	0/2
Seagrave et al. (2014) [92]	0/3	0/3	0/3	0/3	0/3	0/3	0/3	0/3	0/3
Stafford and Norris (2010) [93]	0/27	0/27	0/27	0/27	0/27	0/27	0/27	0/27	0/27
Streubel et al. (2010) [70]	0/97	0/97	0/97	0/97	16/97	0/97	0/97	9/97	25/97
Volgas et al. (2010) [75]	0/10	0/10	0/10	0/10	1/10	0/10	0/10	0/10	1/10
Walker et al. (2019) [94]	0/8	0/8	0/8	0/8	0/8	0/8	0/8	0/8	0/8
Waterman et al. (2017) [53]	0/24	0/24	2/24	1/24	0/24	0/24	0/24	0/24	3/24
Zalavras et al. (2007) [54]	0/11	0/11	0/11	0/11	0/11	0/11	0/11	0/11	0/11
Total (no/total no)	**21/1834**	**13/1834**	13/1834	10/1834	29/1834	0/1834	4/1834	15/1834	105/1834
	**34/1834**								

### Quality assessment

**Fig. 3:**
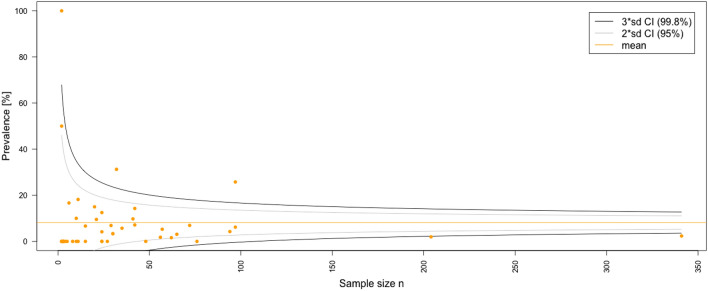
Funnel plot for the overall prevalence of complications

**Fig. 4:**
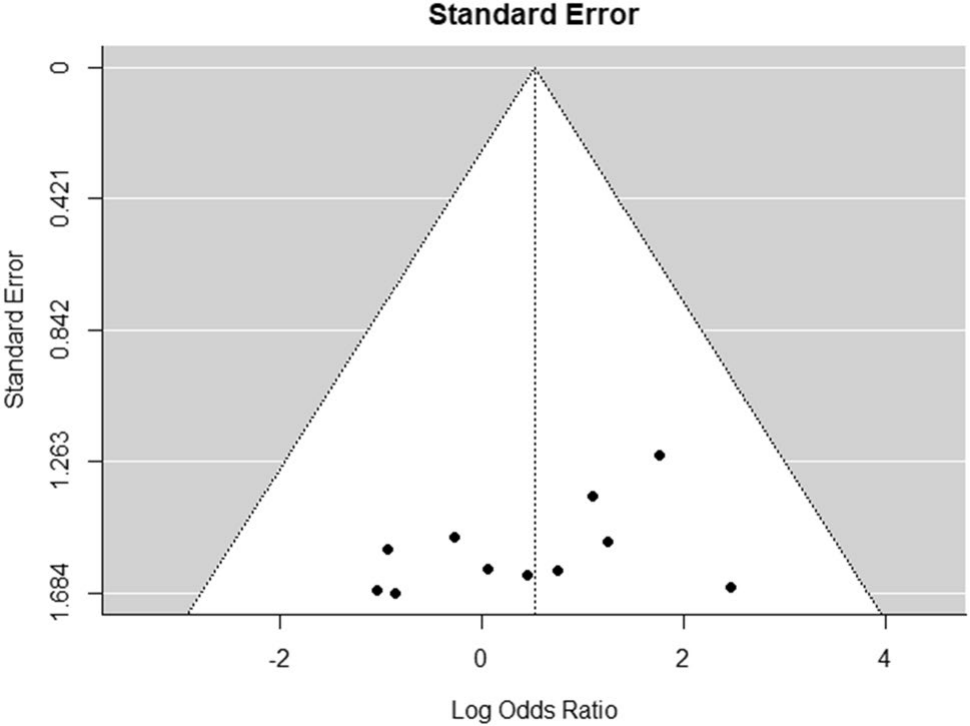
Funnel plot for complications reported in both the tibia and the femur

The results of the critical appraisal of the methodologic quality of all included articles can be found in Table 3. Using JBI’s critical appraisal tools, the average score for the case series (*n* = 35) was 4.4/10 (44%), for the cohort studies (*n* = 9) 7.2/11 (66%) and for the randomized controlled trials (RCT) (*n* = 3) 9.7/13 (75%). The quality assessment using JBI critical appraisal tools revealed that, the overall 62% of the criteria were met. The funnel plot for the overall prevalence of complications detected good symmetrical distribution of the referral points. The majority of the values are close to the no-effect line and very few are outside the CI range of 95%. Overall, poor data dispersion was apparent, indicating a rather low risk of publication bias (Fig. 3). Similarly, no asymmetry in the funnel plot of intervention effect estimates from individual studies versus a measure of individual study size was observed for the comparison of complications in the tibia and femur and, therefore, potential publication bias of the studies can be considered low (Fig. 4).

**Table 3:** Quality analysis using Joanna Briggs Institute critical appraisal tools [28, 39, 40]

	Case series												
Authors/Year of publication	Q1	Q2	Q3	Q4	Q5	Q6	Q7	Q8	Q9	Q10			Total yes (%)
Conway et al. (2014) [77]	Y	Y	U	U	N	Y	N	Y	N	N/A			4/10 (40)
Eisenstein et al. (2016) [79]	Y	Y	N	Y	Y	N	N	N	N	N/A			4/10 (40)
Stafford and Norris (2010) [93]	Y	Y	U	Y	Y	Y	N	Y	N/A	N			6/10 (60)
Waterman et al. (2017) [53]	Y	N	U	Y	Y	Y	N	N	U	N			4/10 (40)
Walker et al. (2019) [94]	Y	Y	U	Y	Y	Y	Y	Y	N	N/A			7/10 (70)
Streubel et al. (2010) [70]	Y	Y	Y	Y	Y	Y	N	Y	N	Y			7/10 (70)
Seagrave et al. (2014) [92]	N	N	N	N	N	Y	N	Y	N	N/A			2/10 (20)
Donders et al. (2016) [18]	N	N	N	N	N/A	Y	N	Y	N	N/A			2/10 (20)
Han et al. (2015) [42]	Y	U	U	Y	Y	N	Y	Y	N	N			5/10 (50)
Herscovici and Scaduto (2012) [80]	N	U	U	N	N	Y	N	U	N	N			1/10 (10)
Jakma et al. (2014) [81]	Y	Y	U	Y	Y	Y	N	Y	U	N			6/10 (60)
Kanakaris et al. (2011) [67]	Y	Y	Y	Y	Y	Y	N	N	N	N/A			6/10 (60)
Kanakaris et al. (2014) [44]	Y	Y	Y	Y	Y	Y	N	Y	N	N/A			7/10 (70)
Qvick et al. (2013) [52]	Y	N	N	Y	Y	Y	N	Y	U	N/A			5/10 (50)
O’Callaghan et al. (2019) [59]	N	U	Y	U	N	Y	Y	Y	N	N/A			4/10 (40)
Niikura et al. (2021) [50]	Y	Y	U	Y	Y	Y	N	N	U	N			5/10 (50)
Newman et al. (2008) [89]	Y	N	N	N	N	Y	N	Y	U	N/A			3/10 (30)
Naruka et al. (2019) [88]	N	Y	Y	U	N/A	Y	Y	Y	U	N/A			5/10 (50)
Moghaddam et al. (2017) [87]	Y	N	Y	Y	Y	Y	Y	Y	U	Y			8/10 (80)
Metsemakers et al. (2019) [86]	Y	N	U	Y	N	Y	N	Y	N	Y			5/10 (50)
McCall et al. (2010) [49]	N	N	Y	Y	U	N	N	Y	N	N			3/10 (30)
Lowe et al. (2011) [25]	N/A	Y	Y	U	U	Y	N	N	N	N/A			3/10 (30)
Lehman et al. (2012) [84]	N/A	Y	N	U	U	Y	N	Y	N/A	N/A			3/10 (30)
Kusnezov et al. (2017) [83]	Y	U	U	Y	Y	Y	N	Y	U	N			5/10 (50)
Krappinger et al. (2015) [82]	N	Y	Y	U	U	N	N	Y	U	N/A			3/10 (30)
Grote et al. (2015) [57]	Y	N	Y	Y	U	N	N	N	N	N			3/10 (30)
Cipriano et al. (2012) [23]	Y	Y	Y	Y	U	N	N	Y	U	N			5/10 (50)
Barlow and Kuhn (2014) [55]	N	Y	Y	U	U	N	N	Y	N	N			3/10 (30)
Lowe et al. (2010) [46]	N	Y	Y	U	U	Y	N	Y	N	N/A			4/10 (40)
Mansour and Conway (2015) [85]	N	Y	Y	U	U	Y	N	Y	U	N/A			4/10 (40)
Quintero et al. (2010) [60]	N	Y	N	U	U	Y	N	Y	N	N			3/10 (30)
Rauck et al. (2016) [91]	N	Y	N	U	U	Y	N	Y	N	N/A			3/10 (30)
Haubruck et al. (2018) [43]	Y	Y	Y	Y	Y	Y	N	Y	N	Y			8/10 (80)
Zalavras et al. (2007) [54]	Y	Y	N	Y	N	Y	N	Y	U	N			5/10 (50)
Marko et al. (2016) [58]	N	Y	N	U	U	Y	Y	Y	N	N/A			4/10 (40)
											Subtotal (%)		4.4/10 (44)

	Cohort studies												
Authors/Year of publication	Q1	Q2	Q3	Q4	Q5	Q6	Q7	Q8	Q9	Q10	Q11		Total yes (%)
Belthur et al. (2008) [41]	Y	Y	Y	N	N	Y	Y	Y	Y	Y	Y		9/11 (82)
Calori et al. (2014) [56]	Y	U	Y	N	N	Y	Y	Y	Y	N/A	N		6/11 (55)
Davis et al. (2015) [78]	Y	Y	Y	Y	Y	Y	Y	Y	U	N/A	Y		9/11 (82)
Onsea et al. (2021) [51]	N	Y	Y	U	U	N/A	Y	Y	U	N/A	Y		5/11 (45)
Nodzo et al. (2014) [90]	Y	N	Y	Y	Y	Y	Y	Y	Y	Y	Y		10/11 (91)
Moghaddam et al. (2015) [74]	Y	N	Y	N	U	Y	Y	Y	Y	N	Y		7/11 (64)
Le Baron et al. (2019) [45]	N	Y	Y	Y	N	Y	N	U	N	N	Y		5/11 (45)
Marchand et al. (2017) [47]	U	Y	Y	N	N/A	Y	Y	Y	Y	N/A	Y		7/11 (64)
Martella et al. (2021) [48]	Y	Y	Y	N	N/A	U	Y	Y	Y	N/A	Y		7/11 (64)
												Subtotal (%)	7.2/11 (66)

	Randomized controlled trials																							
Authors/Year of publication	Q1	Q2		Q3		Q4		Q5		Q6		Q7		Q8		Q9		Q10		Q11		Q12	Q13	Total yes (%)
Dawson et al. (2014) [14]	U	U		Y		N		Y		N		Y		N		Y		Y		Y		Y	Y	8/13 (62)
Volgas et al. (2010) [75]	Y	Y		Y		U		N		Y		Y		Y		Y		Y		Y		Y	Y	11/13 (85)
Hall et al. (2017) [69]	Y	Y		Y		N		N		Y		Y		U		Y		Y		Y		Y	Y	10/13 (77)
																						Subtotal (%)		9.7/13 (75)
																						Total		62%

*Y* yes, *N* no, *N/A* not applicable, *U* unknown

### Pooled estimates complication rate

**Fig. 5:**
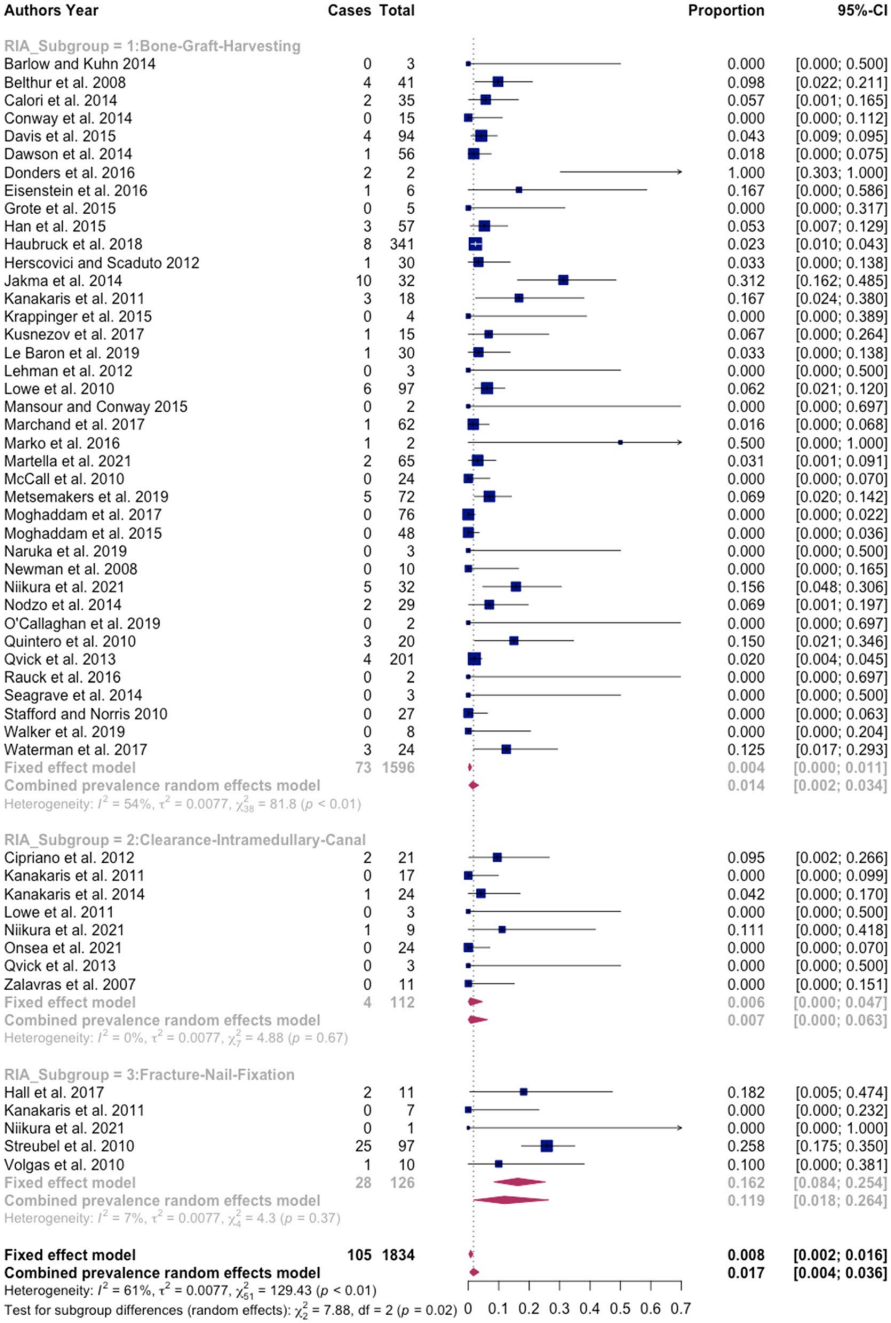
Forest plot for total complication prevalence

Studies included in the proportional meta-analysis for the total complication prevalence were of moderate heterogeneity, as determined by the *I*^2^ statistics (61%). Unexplained heterogeneity within subgroups was moderate in the first subgroup (*I*^2^ = 54%) and low in the second (*I*^2^ = 0%) and third (*I*^2^ = 7%) subgroups. Therefore, the validity of the effect estimates for subgroup 1 is uncertain, as individual trial results are inconsistent. However, overall, sufficient evenly distributed trials for the subgroup analysis to produce meaningful results were observed. Applying meta-analyses of (weighted) average proportion using a random effects model, the pooled estimate of the overall rate of complications associated with RIA system application was 1.7% (95% CI 0.40–3.60). Using the random effects model for subgroup 1, the pooled estimate of the total complication rate was 1.4% (95% CI 0.20–3.40). Among those procedures performed in subgroup 2, the pooled estimate of the rate of total complications was 0.7% (95% CI 0.00–6.30). The pooled estimate of the overall complication rate of RIA system procedures in subgroup 3 was 11.9% (95% CI 1.80–26.40). The test for subgroup differences suggests that there is a statistically significant subgroup effect (*p* = 0.02). Comparing the three subgroups, a higher combined prevalence rate of complications in subgroup 3 (11.9%) compared with subgroup 1 (1.4%) and subgroup 2 (0.7%) was observed. Very low complication rates of the individual outcome measures impaired reliability of the subgroup analyses using the random effects model. A forest plot for the total number of complications was constructed to visualize the estimates with their CIs (Fig. 5). Additional forest plots for the individual outcome measures can be found in Supplementary Material 6.

**Fig. 6:**
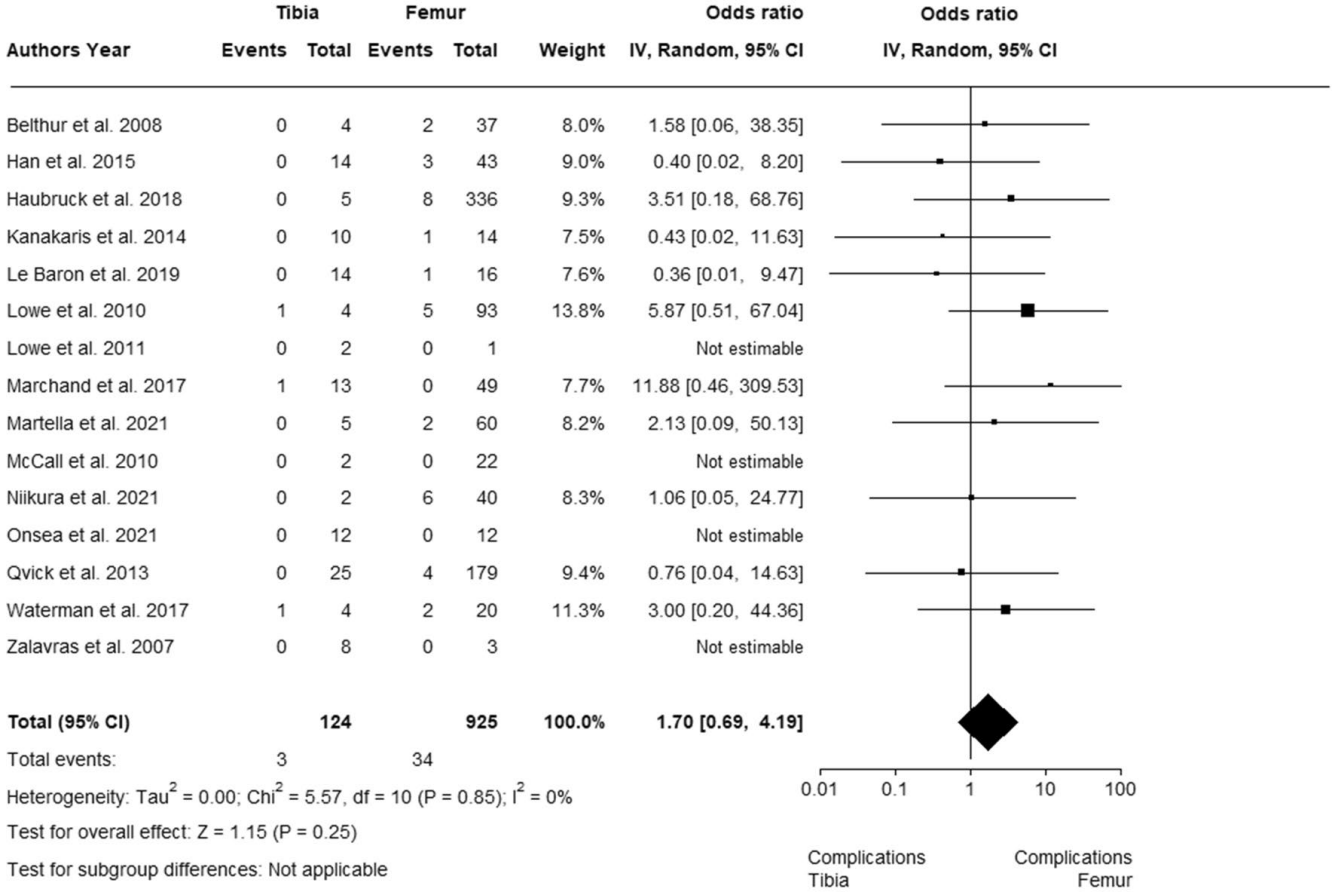
Forest plot for odds ratio comparing tibia and femur using the RIA system

In 15 studies [25, 41–54], the results were described for applications in which the RIA system was used in either femur or tibia in individual patients. The studies included in the meta-analysis comparing the estimated complication rate when the RIA system was applied to the tibia compared with the femur had low heterogeneity, as determined by the I^2^ statistic (0%). Using the random effects model, the total OR of 1.7 (95% CI 0.69–4.19) was nonsignificant in favor of the femur associated with more overall complications (Fig. 6).

### Blood loss

Thirteen studies [18, 42, 43, 45, 47, 48, 50, 55–60] reported blood loss in a total number of 336 performed procedures with the RIA system. Of these 13 studies, five studies [47, 48, 50, 56, 58] reported the volume of blood lost, which resulted in a mean of 803.29 ml per case. Furthermore, five out of the 13 studies reported a drop in hemoglobin, resulting in a mean of 3.74 g/dl. In total, 60 out of 583 patients (9.72%) included in the studies [18, 42, 43, 45, 47, 48, 57, 59, 60] required blood transfusion. The findings on blood loss are summarized in Table 4.

**Table 4:** Findings on blood loss associated with using the RIA system

Authors/Year of publication	Total number of procedures assessed	Blood loss		Blood transfusion (no/total no)
		Volume (ml)	Drop in hemoglobin (g/dl)	
Barlow and Kuhn (2014) [55]	3	‘No excessive blood loss’		
Calori et al. (2014) [56]	35	572.85	1.66	–
Donders et al. (2016) [18]	2	–	4.40	2/2
Grote et al. (2015) [57]	5	–	3.30	0/5
Han et al. (2015) [42]	57	–	3.15	7/57
Haubruck et al. (2018) [43]	11	–	6.20	11/341
Le Baron et al. (2019) [45]	30	–	–	3/30
Marchand et al. (2017) [47]	62	647.00	–	27/62
Marko et al. (2016) [58]	2	1250.00	–	–
Martella et al. (2021) [48]	65	388.00	–	9/64
Niikura et al. (2021) [50]	42	1158.60	–	–
O’Callaghan et al. (2019) [59]	2	–	–	0/2
Quintero et al. (2010) [60]	20	–	–	1/20
Total	336	803.29	3.74	60/583 (9.72%)

## Discussion

To date, neither the complications of the RIA system in ABG harvesting nor in the alternative applications of clearance of the intramedullary canal and nail fixation of long bone fractures have been systematically investigated. Yet, it is imperative to have knowledge of the complication prevalence associated with the RIA system in order to achieve optimal informed patient consent and to optimize perioperative management and intraoperative use of the device.

The meta-analysis with weighted average proportions for RIA system application revealed an overall low prevalence of complications (1.7%), which is in line with a recent narrative review [61]. A considerable aspect of the RIA system is the possibility of harvesting large quantities of bone graft from the femur, whereby especially an extra-articular approach via the greater trochanter is possible, while the semi-elastic plastic nitinol unit is aligned with the mid-axis of the femur [52]. However, particularly in the tibia, application of the original RIA 1 device (smallest available reamer size of 12 mm diameter) can be technically challenging due to an eccentric starting point with a relative rigid tip and semi-flexible shaft-tubing system. Although for the femur cadaveric studies have shown no significant interference with the biomechanical properties of the harvested femur [62, 63], no such in vitro biomechanical data are available for the tibia as harvesting site. Therefore, the observed tendency of higher complication rates in the femur described in the present meta-analysis (OR 1.7; 95% CI 0.69–4.19) appears, at first glance, to be counterintuitive. However, this result could be influenced by the design of primary research studies, such as the largest study [52], in which the RIA system was applied to the same side of the lower extremity for tibial index surgeries and to the contralateral side in femoral index surgeries. Therefore, the increased complication rate observed when using the RIA system in the femur could be associated with the postoperative weight bearing of the harvested femur, whereas in the harvested tibia, the weight bearing of the extremity was restricted.

Noteworthy, the RIA 2 system, launched in 2020, includes exchangeable reamer heads with the smallest diameter of 10 mm. Early data for RIA 2 system applied in cadaveric femur and tibia, however, indicates that it should be used with caution in the tibia because of increased perforation risk [64]. Clinical data of RIA 2 system applications have not yet been published. Nonetheless, taken together, technical or surgical error such as eccentric reaming and cortical thinning are of importance as they seem to be one of the major reasons for intra- and postoperative complications associated with using the RIA system [52]. Therefore, while intraoperative diligence and fluoroscopic monitoring are paramount to decrease the risk of technical errors and complications, it is important to note that surgeons who frequently use the RIA system describe a steep learning curve in the use of the system [43, 65]. To shorten the learning curve and reduce complications, special training at a ‘center of excellence’ is recommended [43].

In cases of osteomyelitis or peri-implant infection in the long bones of the lower extremity, the entire length of the medullary canal must be debrided, and the RIA system can be used instead of conventional reamers to take advantage of its additional irrigation and aspiration function [66]. Aggressive (over)reaming is not required compared to those cases in which harvesting large bone graft volumes is the goal. The treatment of osteomyelitis or peri-implant infection are scheduled as elective procedures. Thus, very low prevalence of complications of 0.7% (95% CI 0.0–6.3) is expected and makes the application of the RIA system for this indication appealing. The advantage of minimizing intramedullary pressure and potential intravasation of, for example, tumor cells, as well as obtaining multiple samples for further histological analysis, argue for the use in bone malignancy cases [23, 67]. Further, next to debulking of intramedullary neoplasm, in a pilot study with three cases illustrated by Lowe et al. (2011) [25], by profiting from the sharp, front-end cutting reamer heads, the RIA system showed the capacity to remove debonded cement fragments when traditional removal methods such as intramedullary hooks, reverse curettes, flexible osteotomes or stacked guide rods [68] failed.

For intramedullary reaming during treatment of femoral shaft fractures no clinically relevant protective effects of the RIA system are reported in nonpolytrauma patients [69], while there might be a potential advantage in polytrauma patients [67]. In line with the results of the present meta-analysis of increased complication rate for the subgroup of RIA system reaming before nail fixation (complication prevalence of 11.9%; 95% CI 1.8–26.4), its application for this purpose should be chosen with caution. However, we note that a potential confounding factor causing differences in treatment effect between the subgroups might have been that this group consists of acute fracture treatments. In particular, one large study (*n* = 97 cases) in subgroup 3 reported cardiopulmonary complications/systemic infections or wound infections/local infections in 25 cases [70], all of which may have been related to the index injury rather than the use of the RIA system. Thus, in particular, fracture treatment itself is a study characteristic that may be confounded with the covariate of interest; namely, RIA system-associated complications.

Clinically significant blood loss during RIA system application, which is associated with continuous irrigation due to the abundant intramedullary blood supply, was first reported in smaller case series [18, 58] and more recently confirmed in multiple larger studies [43, 47, 50, 71]. In the present systematic literature review, a mean blood loss of 803.29 ml, a drop in hemoglobin of 3.74 mg/dl and required blood transfusion in 9.72% of patients were observed. It should be noted that blood loss, particularly in surgically complex patients, is multifactorial and might be affected by perioperative and postoperative fluid administration regimes; the complexity of the nonunion repair, including the extent of recipient site preparation; and the amount of ABG required [71]. Nonetheless, these findings are paramount for every surgeon, as surgical planning includes interdisciplinary communication and preparation for potential intraoperative blood loss. Patients at a high risk for anemia-associated comorbidities [72] may benefit from intraoperative auto-transfusion of the blood lost due to the RIA system. However, cell saver filter cannot handle the large volume of fluid [58] that is produced when using the RIA system. Therefore, transfusion risk should be discussed with patients, and the choice of the RIA system should be carefully considered in patients with anemia or bleeding risk [71]. Furthermore, reduced blood loss with repeated use of the device by the same surgical team is described [50], potentially by avoiding prolonged suction and aspiration using the RIA system in later stages of the learning curve. This is emphasized by the recently reported results of a pilot study of 24 patients with femoral bone graft harvesting and a mean drop of postoperative hemoglobin as high as 4.1 g/dl as well as the necessity for transfusion of packed red blood cells in 33% of cases [73].

### Limitations

We note several limitations to this study. The retrospective design of most of the included studies is an important limitation. Only ten studies had a prospective study design [14, 44, 45, 49, 53, 57, 67, 69, 74, 75] of which only three were RCTs [14, 69, 75], which represents an important source of selection bias. Furthermore, trials per subgroup for total complications were not evenly distributed. However, valid interpretation of the findings was conducted following the guidelines for improving the interpretation of subgroup analyses in reviews [76]. A complication with more than ten events out of the total 1834 procedures was considered clinically relevant by the authors and, therefore, an additional meta-analysis was performed only in these cases (Supplementary Material 6).

## Conclusions

As demonstrated in this systematic review and meta-analysis, the overall prevalence of complications with the RIA system is low. Yet, in particular, the risk of cortex perforations and commonly reported relevant intraoperative blood loss are complications to be anticipated in the perioperative management and ultimately during the use of the RIA system.

## Supplementary Information

Below is the link to the electronic supplementary material.

Supplementary file1 (DOCX 4490 KB)
