# Supplementary material for: Complications associated using the reamer–irrigator –aspirator (RIA) system: a systematic review and meta-analysis
Source: Arch Orthop Trauma Surg. 2022 Sep 17;143(7):3823–43. doi: 10.1007/s00402-022-04621-z (PMC10293355; doi:10.1007/s00402-022-04621-z)
Supplement: Supplementary file 1 — Supplementary file1 (DOCX 4490 KB) [file 402_2022_4621_MOESM1_ESM.docx]

Supplementary Material

# Supplementary Material 1

PRISMA 2020 Checklist [1]

| **Section and Topic** | **Item #** | **Checklist item** | **Location where item is reported** |
| --- | --- | --- | --- |
| **TITLE** | | |  |
| Title | 1 | Identify the report as a systematic review. | Lines 1-3 |
| **ABSTRACT** | | |  |
| Abstract | 2 | See the PRISMA 2020 for Abstracts checklist. | Lines 4-28 |
| **INTRODUCTION** | | |  |
| Rationale | 3 | Describe the rationale for the review in the context of existing knowledge. | Lines 33-67 |
| Objectives | 4 | Provide an explicit statement of the objective(s) or question(s) the review addresses. | Lines 68-69 |
| **METHODS** | | |  |
| Eligibility criteria | 5 | Specify the inclusion and exclusion criteria for the review and how studies were grouped for the syntheses. | Lines 78-79 |
| Information sources | 6 | Specify all databases, registers, websites, organisations, reference lists and other sources searched or consulted to identify studies. Specify the date when each source was last searched or consulted. | Lines 91-95 |
| Search strategy | 7 | Present the full search strategies for all databases, registers and websites, including any filters and limits used. | Line 95 |
| Selection process | 8 | Specify the methods used to decide whether a study met the inclusion criteria of the review, including how many reviewers screened each record and each report retrieved, whether they worked independently, and if applicable, details of automation tools used in the process. | Lines 97-98 |
| Data collection process | 9 | Specify the methods used to collect data from reports, including how many reviewers collected data from each report, whether they worked independently, any processes for obtaining or confirming data from study investigators, and if applicable, details of automation tools used in the process. | Line 104 |
| Data items | 10a | List and define all outcomes for which data were sought. Specify whether all results that were compatible with each outcome domain in each study were sought (e.g. for all measures, time points, analyses), and if not, the methods used to decide which results to collect. | Lines 107-112 |
|  | 10b | List and define all other variables for which data were sought (e.g. participant and intervention characteristics, funding sources). Describe any assumptions made about any missing or unclear information. | Lines 104-107 |
| Study risk of bias assessment | 11 | Specify the methods used to assess risk of bias in the included studies, including details of the tool(s) used, how many reviewers assessed each study and whether they worked independently, and if applicable, details of automation tools used in the process. | Lines 137-145 |
| Effect measures | 12 | Specify for each outcome the effect measure(s) (e.g. risk ratio, mean difference) used in the synthesis or presentation of results. | Lines 115-116 + lines 119-121 |
| Synthesis methods | 13a | Describe the processes used to decide which studies were eligible for each synthesis (e.g. tabulating the study intervention characteristics and comparing against the planned groups for each synthesis (item #5)). | Lines 114-121 |
|  | 13b | Describe any methods required to prepare the data for presentation or synthesis, such as handling of missing summary statistics, or data conversions. | Lines 120-121 |
|  | 13c | Describe any methods used to tabulate or visually display results of individual studies and syntheses. | Lines 115-116 + lines 119-121 |
|  | 13d | Describe any methods used to synthesize results and provide a rationale for the choice(s). If meta-analysis was performed, describe the model(s), method(s) to identify the presence and extent of statistical heterogeneity, and software package(s) used. | Lines 122-135 |
|  | 13e | Describe any methods used to explore possible causes of heterogeneity among study results (e.g. subgroup analysis, meta-regression). | Lines 130-131 |
|  | 13f | Describe any sensitivity analyses conducted to assess robustness of the synthesized results. | NA |
| Reporting bias assessment | 14 | Describe any methods used to assess risk of bias due to missing results in a synthesis (arising from reporting biases). | Lines 137-145 |
| Certainty assessment | 15 | Describe any methods used to assess certainty (or confidence) in the body of evidence for an outcome. | Lines 130-131 |
| **RESULTS** | | |  |
| Study selection | 16a | Describe the results of the search and selection process, from the number of records identified in the search to the number of studies included in the review, ideally using a flow diagram. | Lines 147-153 |
|  | 16b | Cite studies that might appear to meet the inclusion criteria, but which were excluded, and explain why they were excluded. | Lines 150-151 |
| Study characteristics | 17 | Cite each included study and present its characteristics. | Lines 155-156 |
| Risk of bias in studies | 18 | Present assessments of risk of bias for each included study. | Lines 165-175 |
| Results of individual studies | 19 | For all outcomes, present, for each study: (a) summary statistics for each group (where appropriate) and (b) an effect estimate and its precision (e.g. confidence/credible interval), ideally using structured tables or plots. | Lines 155-163 |
| Results of syntheses | 20a | For each synthesis, briefly summarise the characteristics and risk of bias among contributing studies. | Lines 177-182 |
|  | 20b | Present results of all statistical syntheses conducted. If meta-analysis was done, present for each the summary estimate and precision (e.g. confidence/credible interval) and measures of statistical heterogeneity. If comparing groups, describe direction of the effect. | Lines 182-201 |
|  | 20c | Present results of all investigations of possible causes of heterogeneity among study results. | NA |
|  | 20d | Present results of all sensitivity analyses conducted to assess the robustness of the synthesized results. | NA |
| Reporting biases | 21 | Present assessments of risk of bias due to missing results (arising from reporting biases) for each synthesis assessed. | NA |
| Certainty of evidence | 22 | Present assessments of certainty (or confidence) in the body of evidence for each outcome assessed. | Lines 182-188 |
| **DISCUSSION** | | |  |
| Discussion | 23a | Provide a general interpretation of the results in the context of other evidence. | Lines 210-286 |
|  | 23b | Discuss any limitations of the evidence included in the review. | Lines 261-267 |
|  | 23c | Discuss any limitations of the review processes used. | Lines 288-295 |
|  | 23d | Discuss implications of the results for practice, policy, and future research. | Lines 297-300 |
| **OTHER INFORMATION** | | |  |
| Registration and protocol | 24a | Provide registration information for the review, including register name and registration number, or state that the review was not registered. | Line 74 |
|  | 24b | Indicate where the review protocol can be accessed, or state that a protocol was not prepared. | Line 74 |
|  | 24c | Describe and explain any amendments to information provided at registration or in the protocol. | NA |
| Support | 25 | Describe sources of financial or non-financial support for the review, and the role of the funders or sponsors in the review. | NA |
| Competing interests | 26 | Declare any competing interests of review authors. | Line 302 |
| Availability of data, code and other materials | 27 | Report which of the following are publicly available and where they can be found: template data collection forms; data extracted from included studies; data used for all analyses; analytic code; any other materials used in the review. | NA |

# Supplementary Material 2

PRISMA-S Checklist [2]

| **Section/topic** | **Item #** | **Checklist item** | **Location(s) Reported** |
| --- | --- | --- | --- |
| **INFORMATION SOURCES AND METHODS** | | | |
| Database name | 1 | Name each individual database searched, stating the platform for each. | Lines 91-92 |
| Multi-database searching | 2 | If databases were searched simultaneously on a single platform, state the name of the platform, listing all of the databases searched. | NA |
| Study registries | 3 | List any study registries searched. | NA |
| Online resources and browsing | 4 | Describe any online or print source purposefully searched or browsed (e.g., tables of contents, print conference proceedings, web sites), and how this was done. | NA |
| Citation searching | 5 | Indicate whether cited references or citing references were examined, and describe any methods used for locating cited/citing references (e.g., browsing reference lists, using a citation index, setting up email alerts for references citing included studies). | Line 95 +  lines 99-100 |
| Contacts | 6 | Indicate whether additional studies or data were sought by contacting authors, experts, manufacturers, or others. | Line 112 |
| Other methods | 7 | Describe any additional information sources or search methods used. | NA |
| **SEARCH STRATEGIES** | | | |
| Full search strategies | 8 | Include the search strategies for each database and information source, copied and pasted exactly as run. | Line 95 |
| Limits and restrictions | 9 | Specify that no limits were used, or describe any limits or restrictions applied to a search (e.g., date or time period, language, study design) and provide justification for their use. | Lines 86-89 |
| Search filters | 10 | Indicate whether published search filters were used (as originally designed or modified), and if so, cite the filter(s) used. | NA |
| Prior work | 11 | Indicate when search strategies from other literature reviews were adapted or reused for a substantive part or all of the search, citing the previous review(s). | NA |
| Updates | 12 | Report the methods used to update the search(es) (e.g., rerunning searches, email alerts). | NA |
| Dates of searches | 13 | For each search strategy, provide the date when the last search occurred. | Line 93 |
| **PEER REVIEW** | | | |
| Peer review | 14 | Describe any search peer review process. | NA |
| **MANAGING RECORDS** | | | |
| Total Records | 15 | Document the total number of records identified from each database and other information sources. | Line 152 |
| Deduplication | 16 | Describe the processes and any software used to deduplicate records from multiple database searches and other information sources. | Lines 96-97 |

# Supplementary Material 3

Search strategies performed on August 10, 2021

**
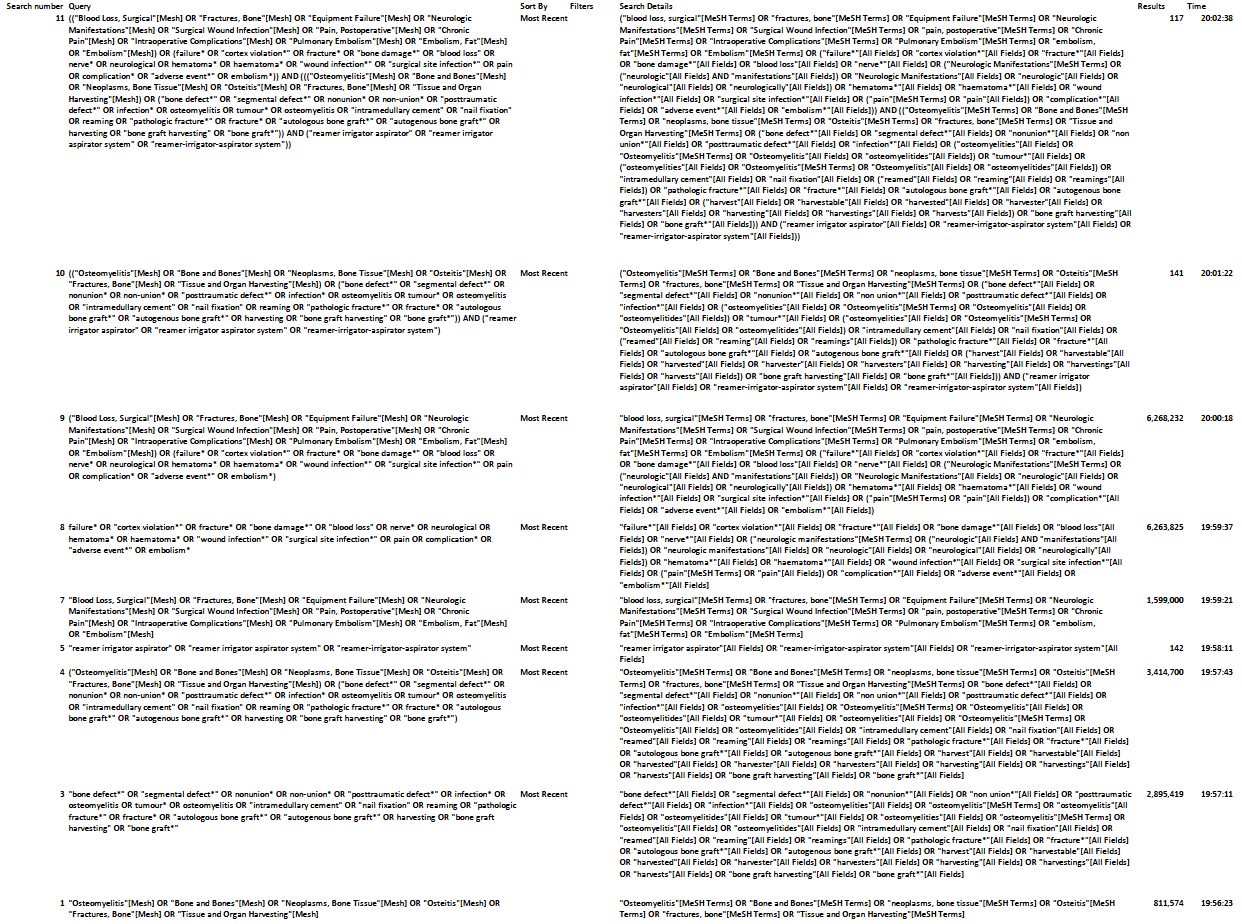

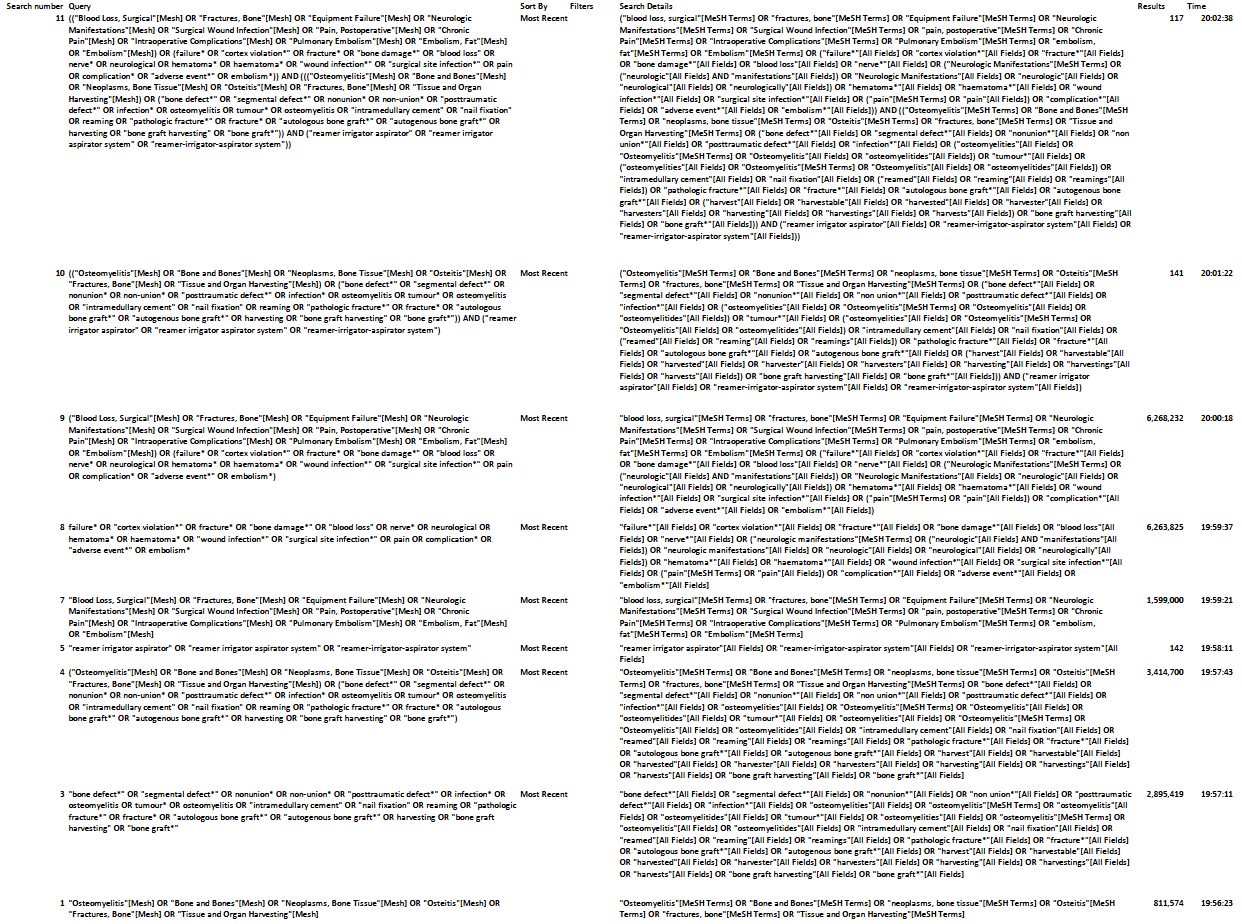
MEDLINE (via PubMed)**


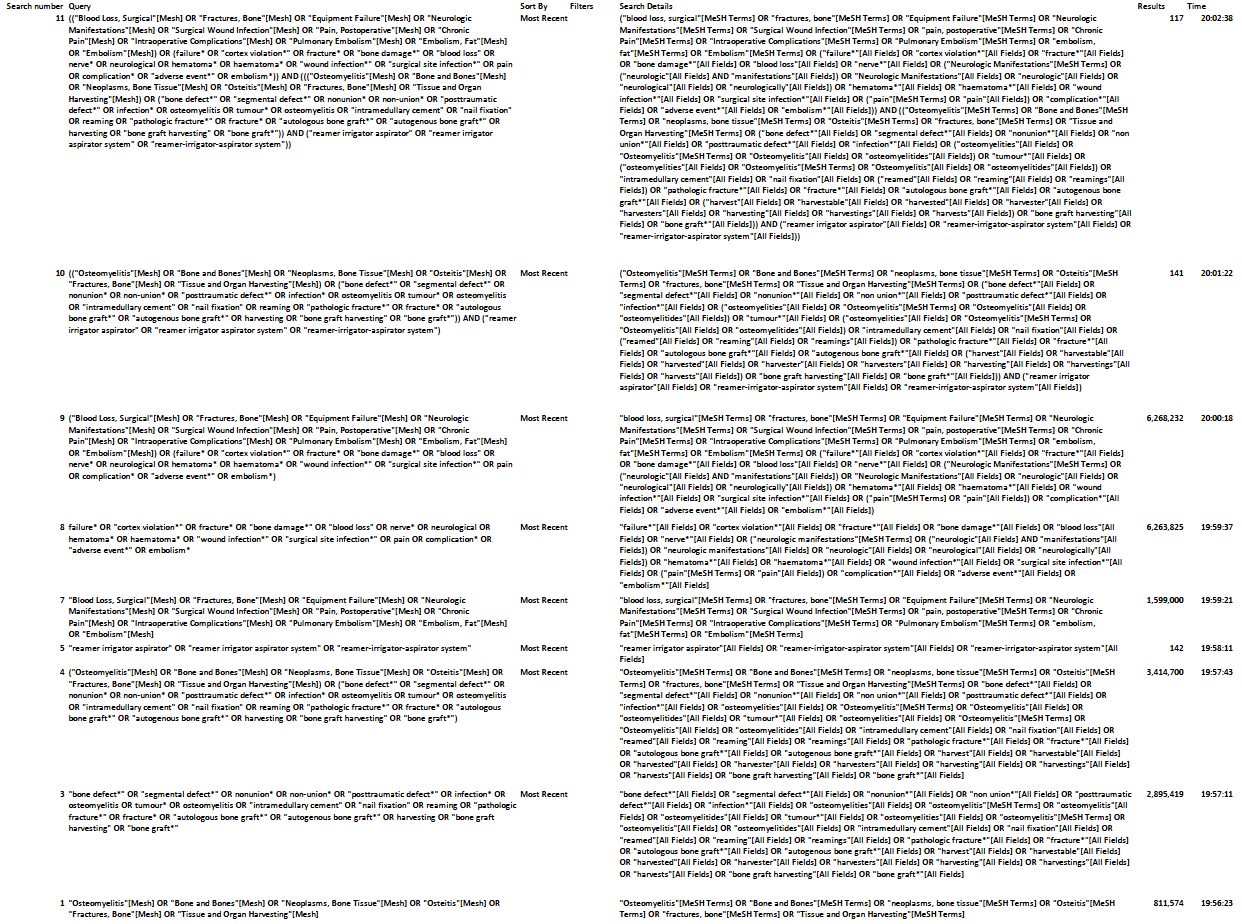


**
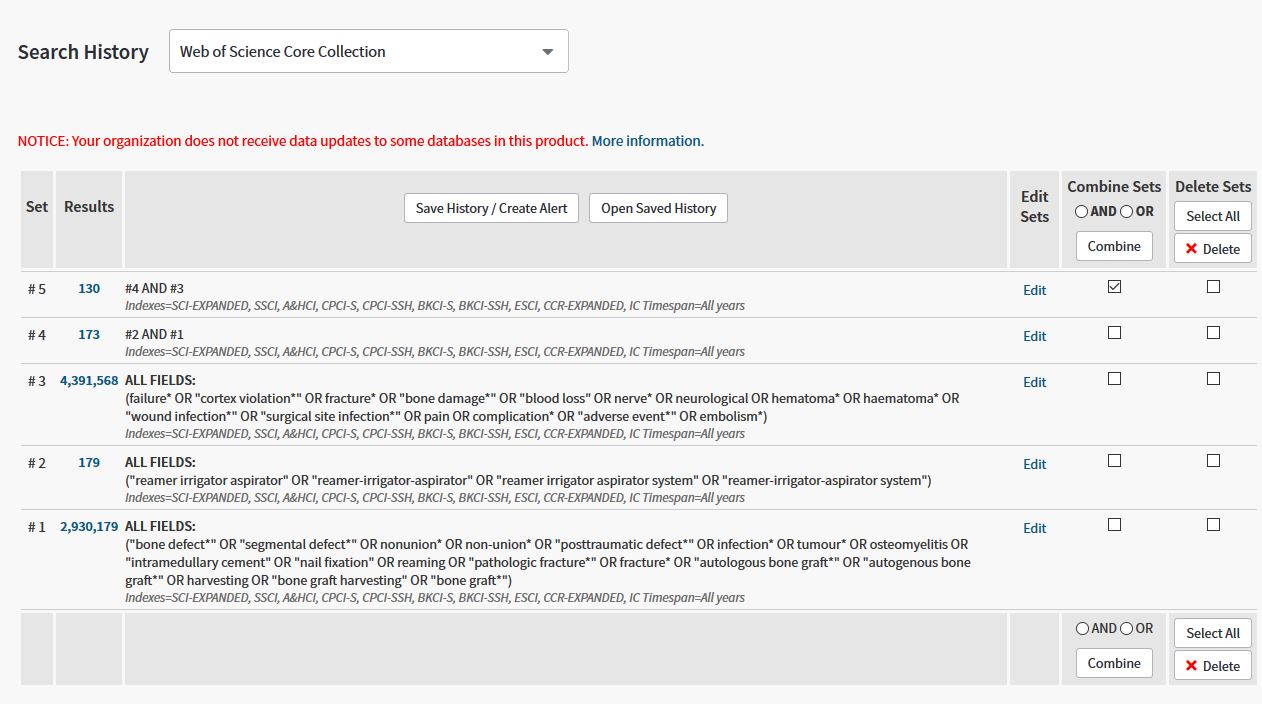
Web of Science Core Collection (Clarivate Analytics)**

**Embase (via Elsevier)**

**
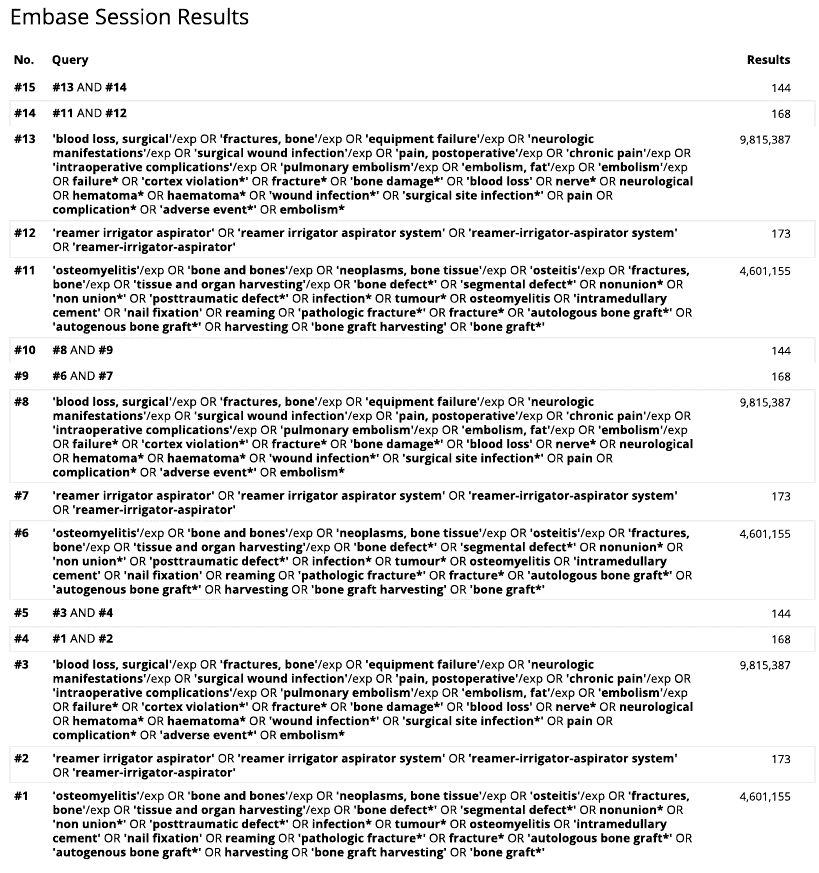
**

# Supplementary Material 4

Joanna Briggs Institute Critical Appraisal Checklists for Case Series, Cohort Studies and Randomized Controlled Trials

| **Case Series [3]** | |
| --- | --- |
| **Q1** | Were there clear criteria for inclusion in the case series? |
| **Q2** | Was the condition measured in a standard, reliable way for all participants included in the case series? |
| **Q3** | Were valid methods used for identification of the condition for all participants included in the case series? |
| **Q4** | Did the case series have consecutive inclusion of participants? |
| **Q5** | Did the case series have complete inclusion of participants? |
| **Q6** | Was there clear reporting of the demographics of the participants in the study? |
| **Q7** | Was there clear reporting of clinical information of the participants? |
| **Q8** | Were the outcomes or follow up results of cases clearly reported? |
| **Q9** | Was there clear reporting of the presenting site(s)/clinic(s) demographic information? |
| **Q10** | Was statistical analysis appropriate? |
| **Cohort Studies [4]** | |
| **Q1** | Were the two groups similar and recruited from the same population? |
| **Q2** | Were the exposures measured similarly to assign people to both exposed and unexposed groups? |
| **Q3** | Was the exposure measured in a valid and reliable way? |
| **Q4** | Were confounding factors identified? |
| **Q5** | Were strategies to deal with confounding factors stated? |
| **Q6** | Were the groups/participants free of the outcome at the start of the study (or at the moment of exposure)? |
| **Q7** | Were the outcomes measured in a valid and reliable way? |
| **Q8** | Was the follow up time reported and sufficient to be long enough for outcomes to occur? |
| **Q9** | Was follow up complete, and if not, were the reasons to loss to follow up described and explored? |
| **Q10** | Were strategies to address incomplete follow up utilized? |
| **Q11** | Was appropriate statistical analysis used? |
| **Randomized Controlled Trials [5]** | |
| **Q1** | Was true randomization used for assignment of participants to treatment groups? |
| **Q2** | Was allocation to treatment groups concealed? |
| **Q3** | Were treatment groups similar at the baseline? |
| **Q4** | Were participants blind to treatment assignment? |
| **Q5** | Were those delivering treatment blind to treatment assignment? |
| **Q6** | Were outcomes assessors blind to treatment assignment? |
| **Q7** | Were treatment groups treated identically other than the intervention of interest? |
| **Q8** | Was follow up complete and if not, were differences between groups in terms of their follow up adequately described and analyzed? |
| **Q9** | Were participants analyzed in the groups to which they were randomized? |
| **Q10** | Were outcomes measured in the same way for treatment groups? |
| **Q11** | Were outcomes measured in a reliable way? |
| **Q12** | Was appropriate statistical analysis used? |
| **Q13** | Was the trial design appropriate, and any deviations from the standard RCT design (individual randomization, parallel groups) accounted for in the conduct and analysis of the trial? |

# Supplementary Material 5

Reason for exclusion after full text screening

| **Year of publication** | **Author(s)** | **Title** | **Journal** | **DOI** | **Reason for exclusion** |
| --- | --- | --- | --- | --- | --- |
| 2017 | Attum B., Douleh D., Whiting P. S., White-Dzuro G. A., Dodd A. C., Shen M. S., Mir H. R., Obremskey W. T., Sethi M. K. | Outcomes of distal Femur nonunions treated with a combined nail/plate construct and autogenous bone grafting | J Orthop Trauma | 10.1097/bot.0000000000000926 | Complication rate not assessed |
| 2007 | Bellapianta J., Gerdeman A., Sharan A., Lozman J. | Use of the reamer irrigator aspirator for the treatment of a 20-year recurrent osteomyelitis of a healed femur fracture | J Orthop Trauma | 10.1097/BOT.0b013e318051532d | Case report |
| 2015 | Bićanić G., Crnogaća K., Aljinović A., Dubravčić I.D., Delimar D. | Useful technique for allograft bone harvest | Acta Clinica Croatica |  | Reamer Irrigator Aspirator system not used |
| 2020 | Bourgeois M., Loisel F., Bertrand D., Nallet J., Gindraux F., Adam A., Lepage D., Sergent P., Leclerc G., Rondot T., Garbuio P., Obert L., Pluvy I. | Management of forearm bone loss with induced membrane technique | Hand Surgery and Rehabilitation | 10.1016/j.hansur.2020.02.002 | Complication rate not assessed |
| 2013 | Cadet E. R., Yin B., Schulz B., Ahmad C. S., Rosenwasser M. P. | Proximal humerus and humeral shaft nonunions | Journal of the American Academy of Orthopaedic Surgeons | 10.5435/00124635-201309020-00005 | Review |
| 2010 | Cobbs K. F | RIA use in a community orthopedic trauma practice: Applying technology, respecting biology | Injury | 10.1016/s0020-1383(10)70015-2 | Complication rate not assessed |
| 2016 | Crist B. D., Stoker A. M., Stannard J. P., Cook J. L. | Analysis of relevant proteins from bone graft harvested using the reamer irrigator and aspirator system (RIA) versus iliac crest (IC) bone graft and RIA waste water | Injury | 10.1016/j.injury.2016.05.030 | In vitro study |
| 2010 | Cuttica D. J., DeVries J. G., Hyer C. F. | Autogenous bone graft harvest using reamer irrigator aspirator (RIA) Technique for tibiotalocalcaneal arthrodesis | Journal of Foot and Ankle Surgery | 10.1053/j.jfas.2010.08.003 | Complication rate not assessed |
| 2018 | De Carolis O., Mori C. M., Vicenti G., Carrozzo M., Abate A., Caiaffa V. | A lifelong story: Case report of a humeral shaft nonunion successfully treated after 30 years | Injury | 10.1016/j.injury.2018.11.031 | Case report |
| 2010 | Desai P. P., Bell A. J., Suk M. | Treatment of recalcitrant, multiply operated tibial nonunions with the RIA graft and rh-BMP2 using intramedullary nails | Injury | 10.1016/S0020-1383(10)70013-9 | Complication rate not assessed |
| 2011 | Donegan D. J., Scolaro J., Matuszewski P. E., Mehta S. | staged bone grafting following placement of an antibiotic spacer block for the management of segmental long bone defects | Orthopedics | 10.3928/01477447-20110922-16 | Complication rate not assessed |
| 2013 | Erasmo R., Guerra L., Palmieri D. | Biotechnology in the treatment of delayed unions and non-unions of the femur | Journal of Orthopaedics and Traumatology | 10.1007/s10195-013-0258-7 | Conference abstract |
| 2019 | Finelli C. A., Dos Reis F. B., Fernandes H. A., Dell'Aquila A., Carvalho R., Miki N., Franciozi C., Abdalla R., Salles M. J. C. | Intramedullary reaming modality for management of postoperative long bone infection: A prospective randomized controlled trial in 44 patients | Patient Safety in Surgery | 10.1186/s13037-019-0215-3 | Complication rate not assessed |
| 2010 | Finkemeier C. G., Neiman R., Hallare D. | RIA: One community's experience | Orthopedic Clinics of North America | 10.1016/j.ocl.2009.07.007 | Review |
| 2010 | Giannoudis P. V., Tan H. B., Perry S., Tzioupis C., Kanakaris N. K. | The systemic inflammatory response following femoral canal reaming using the reamer-irrigator-aspirator (RIA) device | Injury | 10.1016/S0020-1383(10)70011-5 | In vitro study |
| 2009 | Giannoudis P. V., Tzioupis C., Green J. | Surgical techniques: How I do it? The Reamer/ Irrigator/ Aspirator (RIA) System | Injury | 10.1016/j.injury.2009.07.070 | Review |
| 2011 | Giori N. J., Beaupre G. S. | Femoral fracture after harvesting of autologous bone graft using a reamer/ irrigator/ aspirator | J Orthop Trauma | 10.1097/BOT.0b013e3181e39bf4 | Case report |
| 2018 | Giotikas D., Tarazi N., Spalding L., Nabergoj M., Krkovic M. | Early results of a modified induced membrane technique in the management of traumatic bone loss in the lower limb: A cohort study | Irish Journal of Medical Science | 10.1007/s11845-018-1861-7 | Conference abstract |
| 2014 | Goff T. A. J., Kanakaris N. K. | Management of infected non-union of the proximal femur: A combination of therapeutic techniques | Injury | 10.1016/j.injury.2014.08.046 | Case report |
| 2016 | Gupta G., Ahmad S., Zahid M., Khan A. H., Sherwani M. K. A., Khan A. Q. | Management of traumatic tibial diaphyseal bone defect by "induced-membrane technique" | Indian J Orthop | 10.4103/0019-5413.181780 | Reamer Irrigator Aspirator system not used |
| 2010 | Hartsock L. A., Barfield W. R., Kokko K. P., Liles L. L., Wind T., Green J., Giannoudis P. V. | Randomized prospective clinical trial comparing reamer irrigator aspirator (RIA) to standard reaming (SR) in both minimally injured and multiply injured patients with closed femoral shaft fractures treated with reamed intramedullary nailing (IMN) | Injury | 10.1016/S0020-1383(10)70018-8 | In vitro study |
| 2018 | Helbig L., Bechberger M., Aldeeri R., Ivanova A., Haubruck P., Miska M., Schmidmaier G., Omlor G. W. | Initial peri- and postoperative antibiotic treatment of infected nonunions: Results from 212 consecutive patients after mean follow-up of 34 months | Therapeutics and Clinical Risk Management |  | Complication rate not assessed |
| 2009 | Huffman L. K., Harris J. G., Suk M. | Using the bi-masquelet technique and reamer-irrigator-aspirator for post-traumatic foot reconstruction | Foot and Ankle International | 10.3113/FAI.2009.0895 | Case report |
| 2018 | Jin Z. C., Cai Q. B., Zeng Z. K., Li D., Li Y., Huang P. Z.. Zheng X. H | Research progress on induced membrane technique for the treatment of segmental bone defect | Zhongguo gu shang = China journal of orthopaedics and traumatology | 10.3969/j.issn.1003-0034.2018.05.018 | Language |
| 2020 | Kobbe P., Laubach M., Hutmacher D. W., Alabdulrahman H., Sellei R. M., Hildebrand F. | Convergence of scaffold-guided bone regeneration and RIA bone grafting for the treatment of a critical-sized bone defect of the femoral shaft | European Journal of Medical Research | 10.1186/s40001-020-00471-w | Case report |
| 2008 | Kobbe P., Tarkin I. S., Frink M., Pape H. C. | Voluminous bone graft harvesting of the femoral marrow cavity for autologous transplantation. An indication for the "Reamer-Irrigator-Aspirator-" (RIA-) technique | Unfallchirurg | 10.1007/s00113-007-1359-7 | Case report |
| 2008 | Kobbe P., Tarkin I. S., Pape H. C | Use of the 'reamer irrigator aspirator' system for non-infected tibial non-union after failed iliac crest grafting | Injury | 10.1016/j.injury.2007.12.020 | Case report |
| 2017 | Kreulen C., Lian E., Giza E. | technique for use of trabecular metal spacers in tibiotalocalcaneal arthrodesis with large bony defects | Foot and Ankle International | 10.1177/1071100716681743 | Complication rate not assessed |
| 2021 | Kubes K., Friedman A., Pyle C., Diaz G., Hargett D. | Management of twenty centimeter segmental bone defect of femoral shaft secondary to infected non-union of fracture using masquelet technique: A case report | International Journal of Surgery Case Reports | 10.1016/j.ijscr.2021.106107 | Case report |
| 2020 | Litvina E. A., Semenistyy A. A. | A case report of extensive segmental defect of the humerus treated with Masquelet technique | Journal of Shoulder and Elbow Surgery | 10.1016/j.jse.2020.03.018 | Case report |
| 2019 | Madison R. D., Nowotarski P. J. | The Reamer-Irrigator-Aspirator in nonunion surgery | Orthopedic Clinics of North America | 10.1016/j.ocl.2019.03.001 | Complication rate not assessed |
| 2014 | Makridis K. G., Theocharakis S., Fragkakis E. M., Giannoudis P. V. | Reconstruction of an extensive soft tissue and bone defect of the first metatarsal with the use of Masquelet technique: A case report | Foot and Ankle Surgery | 10.1016/j.fas.2013.11.006 | Case report |
| 2018 | Martin K. D., Englert C. R., Unangst A. M. | Arthroscopic-assisted tibiotalocalcaneal intramedullary nail arthrodesis with reamer-irrigator-aspirator bone grafting | Techniques in Foot and Ankle Surgery | 10.1097/BTF.0000000000000156 | Review |
| 2012 | Masquelet A. C. Benko P. E., Mathevon H., Hannouche D., Obert L | Harvest of cortico-cancellous intramedullary femoral bone graft using the reamer-irrigator-aspirator (RIA) | Orthopaedics and Traumatology: Surgery and Research | 10.1016/j.otsr.2012.01.003 | Case report |
| 2019 | Masquelet A., Kanakaris N. K., Obert L., Stafford P., Giannoudis P. V. | Bone repair using the Masquelet technique | Journal of Bone and Joint Surgery - American Volume | 10.2106/JBJS.18.00842 | Review |
| 2014 | Massen F., Baumbach S., Volkmer E., Mutschler W., Grote S. | Pathologic fracture of the distal radius in a 25-year-old patient with a large unicameral bone cyst | BMC musculoskeletal disorders | 10.1186/1471-2474-15-202 | Case report |
| 2016 | Mauffrey C., Butler N., Hake M. E | Fabrication of an interlocked antibiotic/cement-coated carbon fiber nail for the treatment of long bone osteomyelitis | J Orthop Trauma | 10.1097/BOT.0000000000000587 | Case report |
| 2016 | Mauffrey C., Hake M. E., Chadayammuri V., Masquelet A. C | Reconstruction of long bone infections using the induced membrane technique: Tips and tricks | J Orthop Trauma | 10.1097/BOT.0000000000000500 | Complication rate not assessed |
| 2014 | Mazzola S., Mazza E., Colombo M., Malagoli E., Calori G. M. | RIA system vs. Iliac crest graft in the treatment of nonunions and bone defect | Journal of Orthopaedics and Traumatology | 10.1007/s10195-014-0314-y | Conference abstract |
| 2011 | Miller M. A., Ivkovic A., Porter R., Harris M. B., Estok Ii D. M., Smith R. M., Evans C. H., Vrahas M. S. | Autologous bone grafting on steroids: Preliminary clinical results. A novel treatment for nonunions and segmental bone defects | International Orthopaedics | 10.1007/s00264-010-1013-9 | Complication rate not assessed |
| 2008 | Nichols T. A., Sagi H. C., Weber T. G., Guiot B. H. | An alternative source of autograft bone for spinal fusion: The femur: Technical case report | Neurosurgery | 10.1227/01.neu.0000317390.21927.ad | Case report |
| 2016 | Niikura T., Lee S. Y., Iwakura T., Kurosaka M. | Low-intensity pulsed ultrasound (LIPUS) treatment for the patient with bone reconstruction by the Masquelet technique using RIA | J Orthop Trauma | 10.1097/01.bot.0000489981.32706.91 | Conference abstract |
| 2019 | Puvanesarajah V., Shafiq B., Ingari J. V. | Glenohumeral arthrodesis with Reamer-Irrigator-Aspirator (RIA) bone grafting after traumatic proximal humerus bone loss and flail shoulder | Techniques in hand & upper extremity surgery | 10.1097/BTH.0000000000000226 | Case report |
| 2015 | Rankine J. J., Hodgson R. J., Tan H. B., Cox G., Giannoudis P. V. | MRI appearances of the femur following bone graft harvesting using the Reamer-Irrigator-Aspirator | Injury | 10.1016/S0020-1383(15)30057-7 | Complication rate not assessed |
| 2012 | Sagi H. C., Young M. L., Gerstenfeld L., Einhorn T. A., Tornetta P. | Qualitative and quantitative differences between bone graft obtained from the medullary canal (with a Reamer/Irrigator/Aspirator) and the iliac crest of the same patient | J Bone Joint Surg Am | 10.2106/jbjs.L.00159 | In vitro study |
| 2018 | Sasaki G., Watanabe Y., Miyamoto W., Yasui Y., Morimoto S., Kawano H. | Induced membrane technique using beta-tricalcium phosphate for reconstruction of femoral and tibial segmental bone loss due to infection: technical tips and preliminary clinical results | International Orthopaedics | 10.1007/s00264-017-3503-5 | Complication rate not assessed |
| 2016 | Schroter S., Ateschrang A., Flesch I., Stockle U., Freude T. | First mid-term results after cancellous allograft vitalized with autologous bone marrow for infected femoral non-union | Wiener Klinische Wochenschrift | 10.1007/s00508-015-0797-4 | Reamer Irrigator Aspirator system not used |
| 2018 | Siboni R., Joseph E., Blasco L.. Barbe C., Bajolet O., Diallo S., Ohl X. | Management of septic non-union of the tibia by the induced membrane technique. What factors could improve results? | Orthopaedics & Traumatology-Surgery & Research | 10.1016/j.otsr.2018.04.013 | Reamer Irrigator Aspirator system not used |
| 2019 | Siebenbürger G., Grabein B., Schenck T., Kammerlander C.Bäcker W., Zeckey C. | Eradication of Acinetobacter baumannii/ Enterobacter cloacae complex in an open proximal tibial fracture and closed drop foot correction with a multidisciplinary approach using the Taylor Spatial Frame®: A case report | European Journal of Medical Research | 10.1186/s40001-019-0360-2 | Case report |
| 2021 | Stillson J. E., Bunch C. M., Thomas A. V., Mjaess N., Dynako J. A., Piscoya A. S., Post J. M., Ratigan B. L., Goldstein Z. H., Walsh M. M. | Pathologic fracture and hardware failure in Streptococcus anginosus femoral osteomyelitis: Case report | Ann Med Surg (Lond) | 10.1016/j.amsu.2021.102478 | Case report |
| 2011 | Tan H. B., Theodorides A. A., Rankine J. J., Kanakaris N. K., Giannoudis P. V. | MRI appearances of the femur following bone graft harvesting using the reamer-irrigator-aspirator system | Injury | 10.1016/S0020-1383(11)70096-1 | Conference abstract |
| 2018 | Taylor B. C., Triplet J. J., Johnson D. B., Sharpe B. D., Sullivan B., Canini C. | Retrograde femoral bone graft acquisition using the reamer-irrigator-aspirator | Journal of Long-Term Effects of Medical Implants | 10.1615/JLongTermEffMedImplants.2018027914 | Complication rate not assessed |
| 2021 | Teuber H., Rauer T., Pape H. C., Allemann F. | Nonunion after an open trimalleolar ankle fracture: an extended clinical course and a novel approach to tibio-talo-calcaneal arthrodesis | Journal of Foot and Ankle Surgery | 10.1053/j.jfas.2020.03.025 | Case report |
| 2016 | Tiefenboeck T. M., Zak L., Bukaty A., Wozasek G. E. | Pitfalls in automatic limb lengthening: First results with an intramedullary lengthening device | Orthopaedics and Traumatology: Surgery and Research | 10.1016/j.otsr.2016.07.004 | Complication rate not assessed |
| 2011 | Tzioupis C., Panteliadis P., Gamie Z., Tsiridis E. | Revision of a nonunited subtrochanteric femoral fracture around a failed intramedullary nail with the use of RIA products, BMP-7 and hydroxyapatite: A case report | Journal of Medical Case Reports | 10.1186/1752-1947-5-87 | Case report |
| 2020 | Andrzejowski P., Masquelet A., Giannoudis P. V. | Induced membrane technique (Masquelet) for bone defects in the distal tibia, foot, and ankle: Systematic review, case presentations, tips, and techniques | Foot Ankle Clin | 10.1016/j.fcl.2020.08.013 | Review |
| 2018 | Peschiera V., Staletti L., Cavanna M., Saporito M., Berlusconi M. | Predicting the failure in distal femur fractures | Injury | 10.1016/j.injury.2018.10.001 | Complication rate not assessed |
| 2016 | Stafford P., Norris B., Dadgar A., Calder M. | femoral bone defects managed with the induced-membrane technique: Our preferred method of treatment | Techniques in Orthopaedics | 10.1097/bto.0000000000000164 | Review |
| 2010 | Zalavras C. G., Sirkin M. | Treatment of long bone intramedullary infection using the RIA for removal of infected tissue: Indications, method and clinical results | Injury | 10.1016/S0020-1383(10)70008-5 | Review |

# Supplementary Material 6

Additional forest plots for RIA system complication prevalence

###
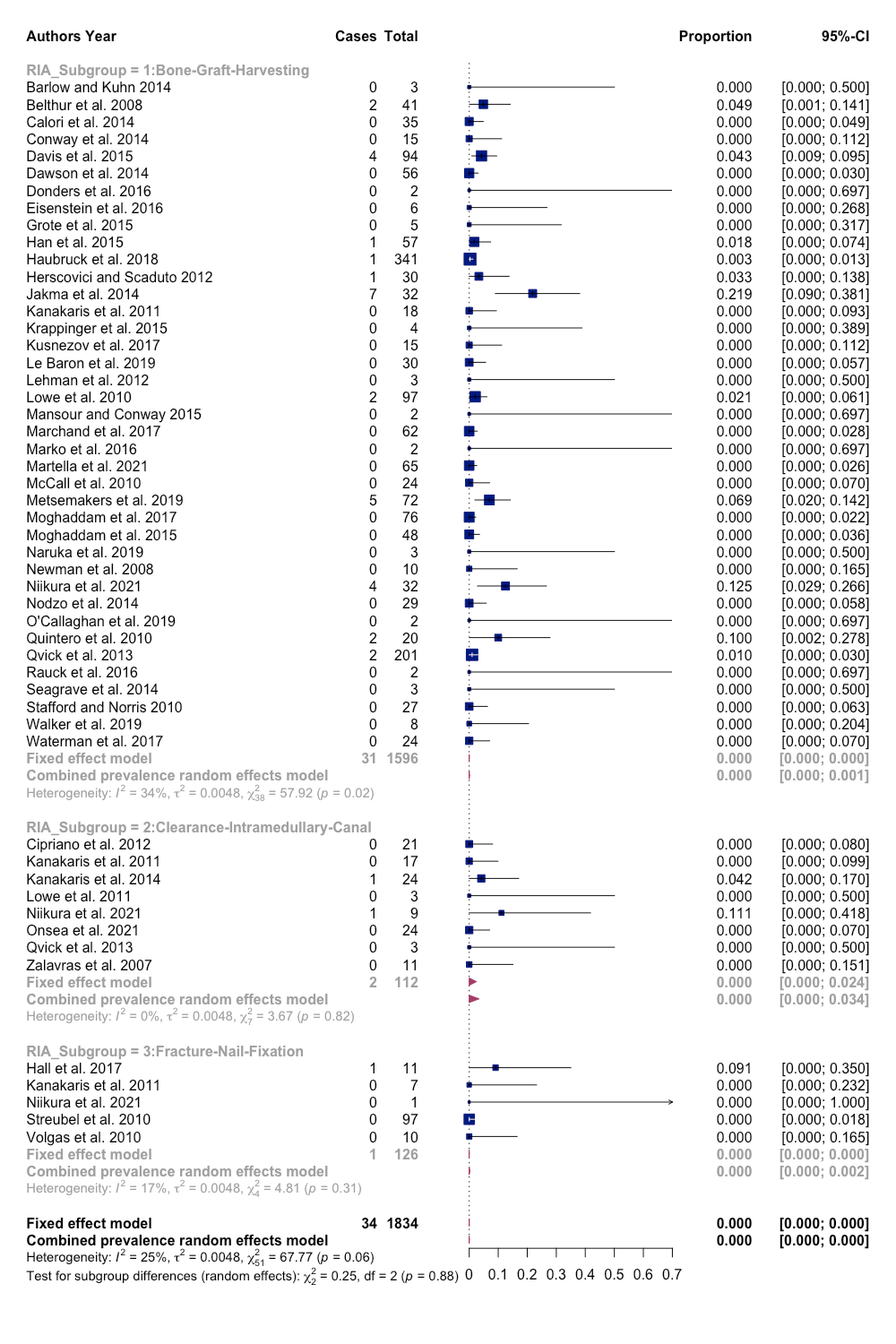
Intraoperative cortex perforations

### Low energy bone fracture during follow-up


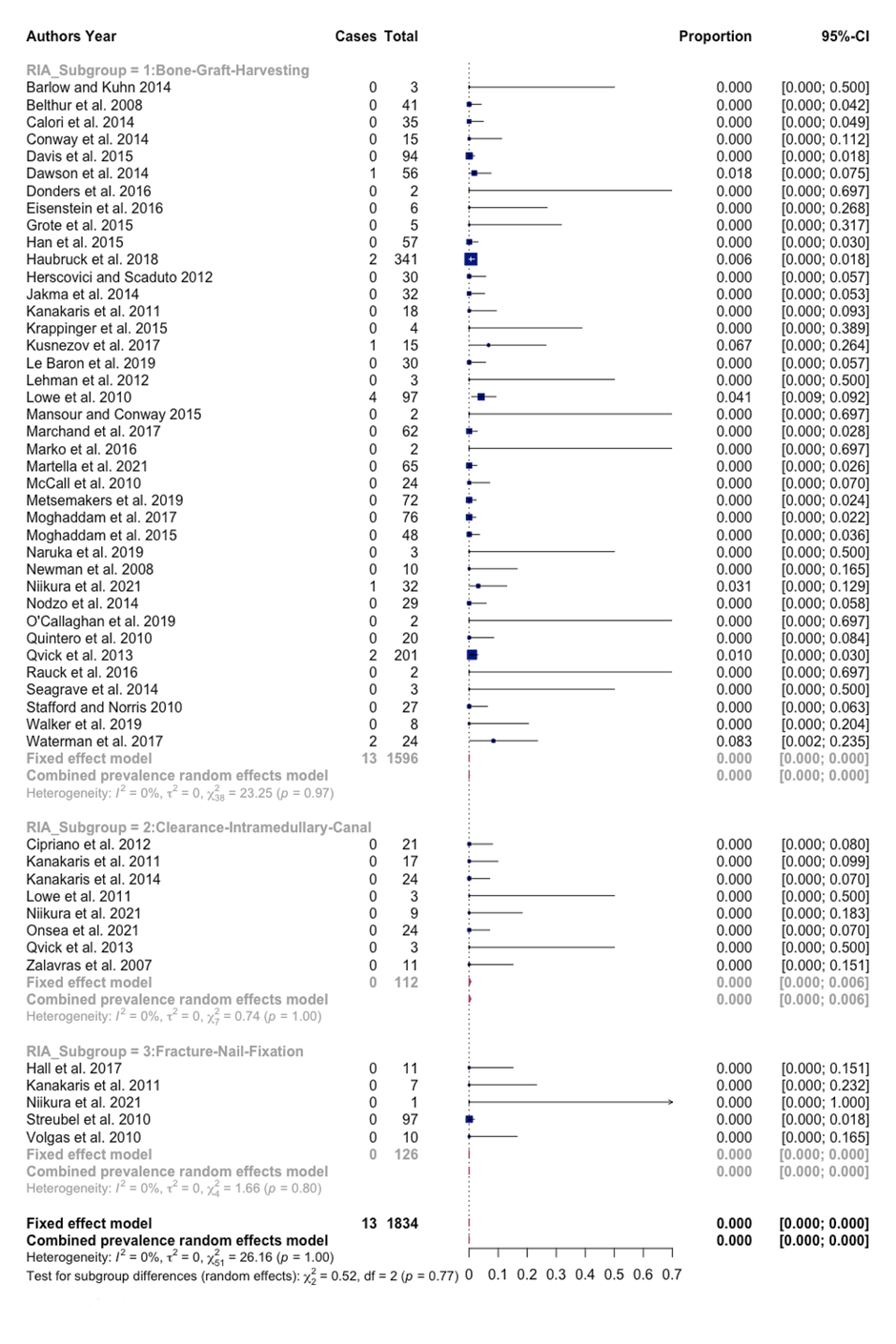


###
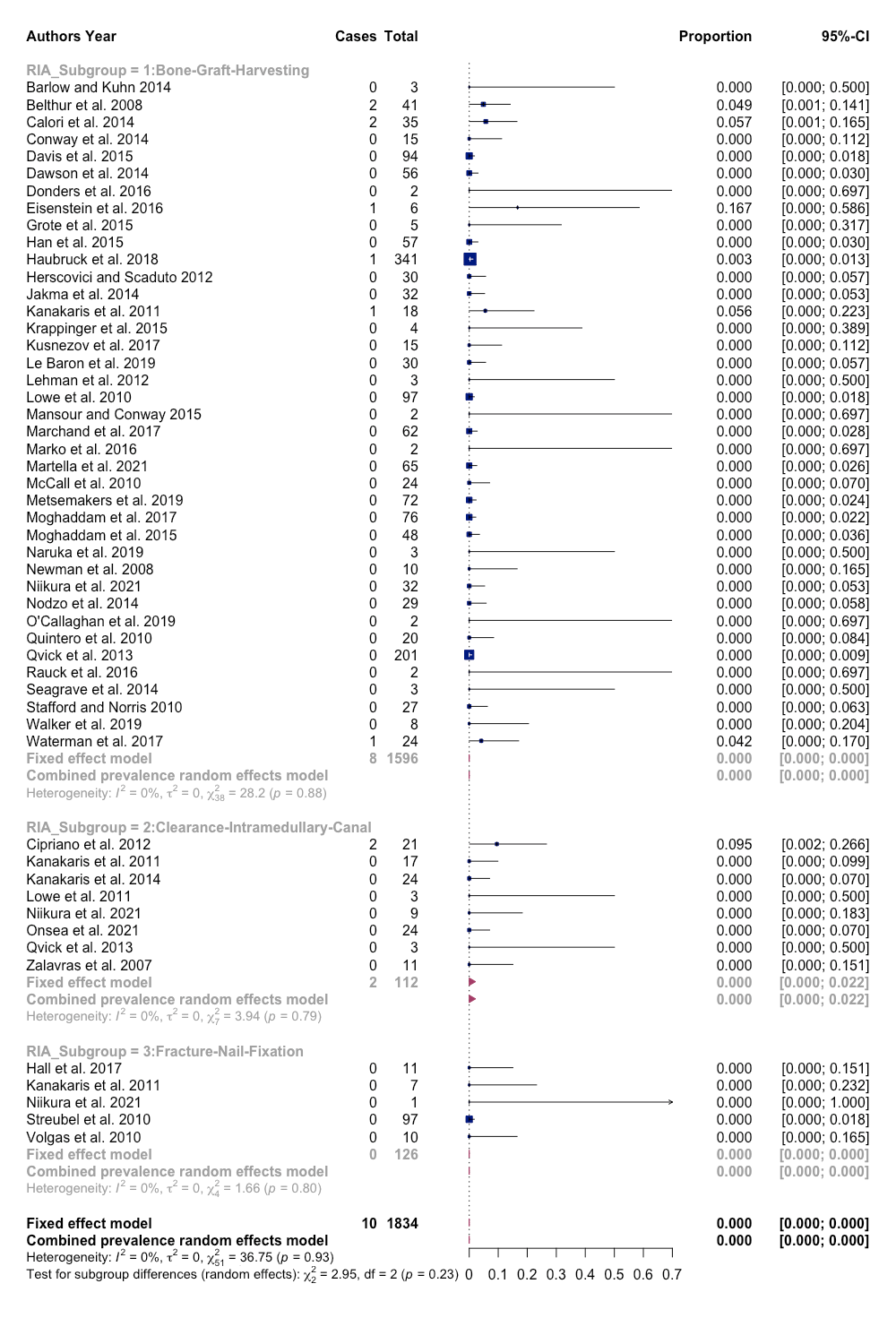
RIA device failure (reamer assembly disengagement, broken tip of the RIA drive shaft or metallic debris)

###
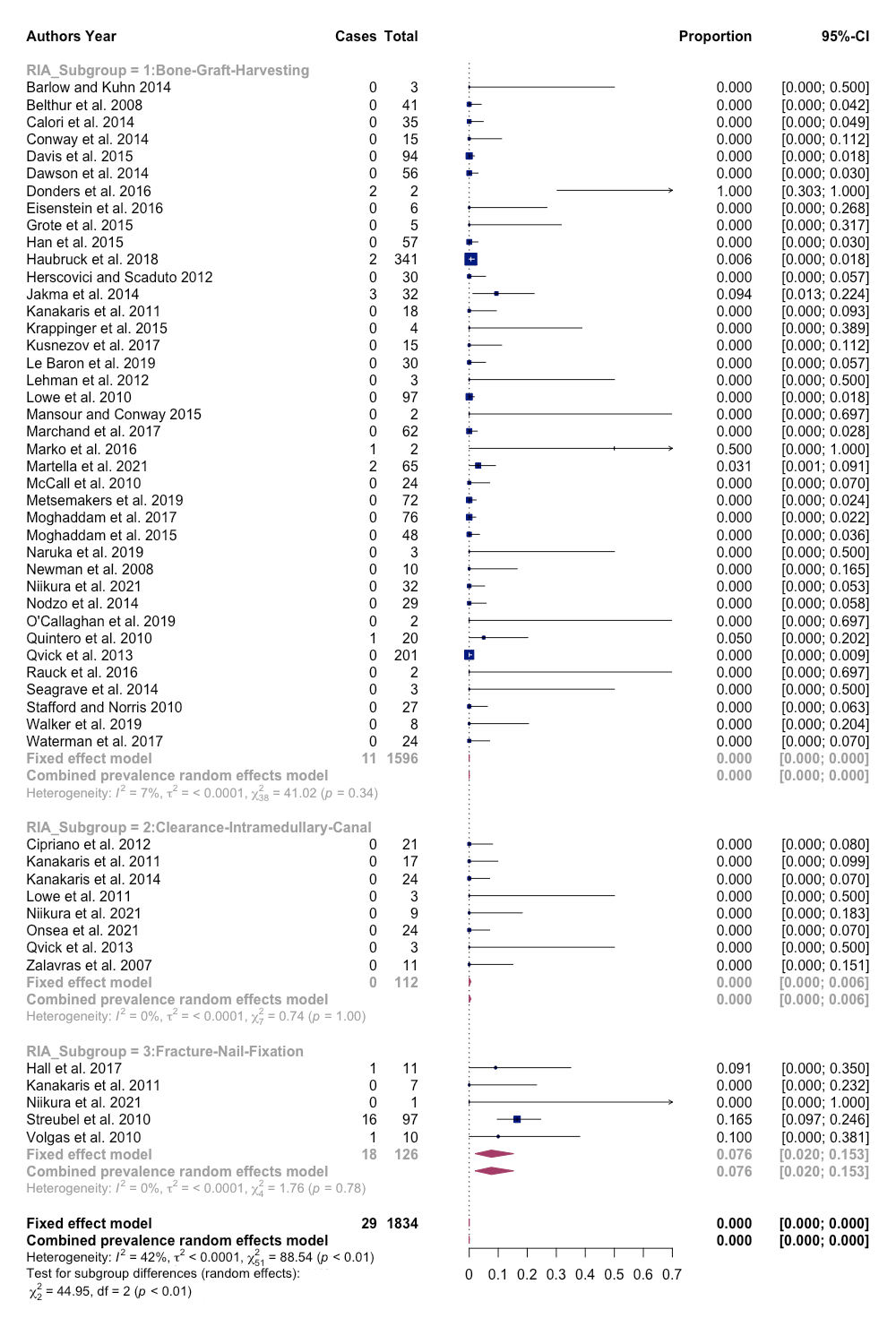
Cardiopulmonary complications / systemic infection

###
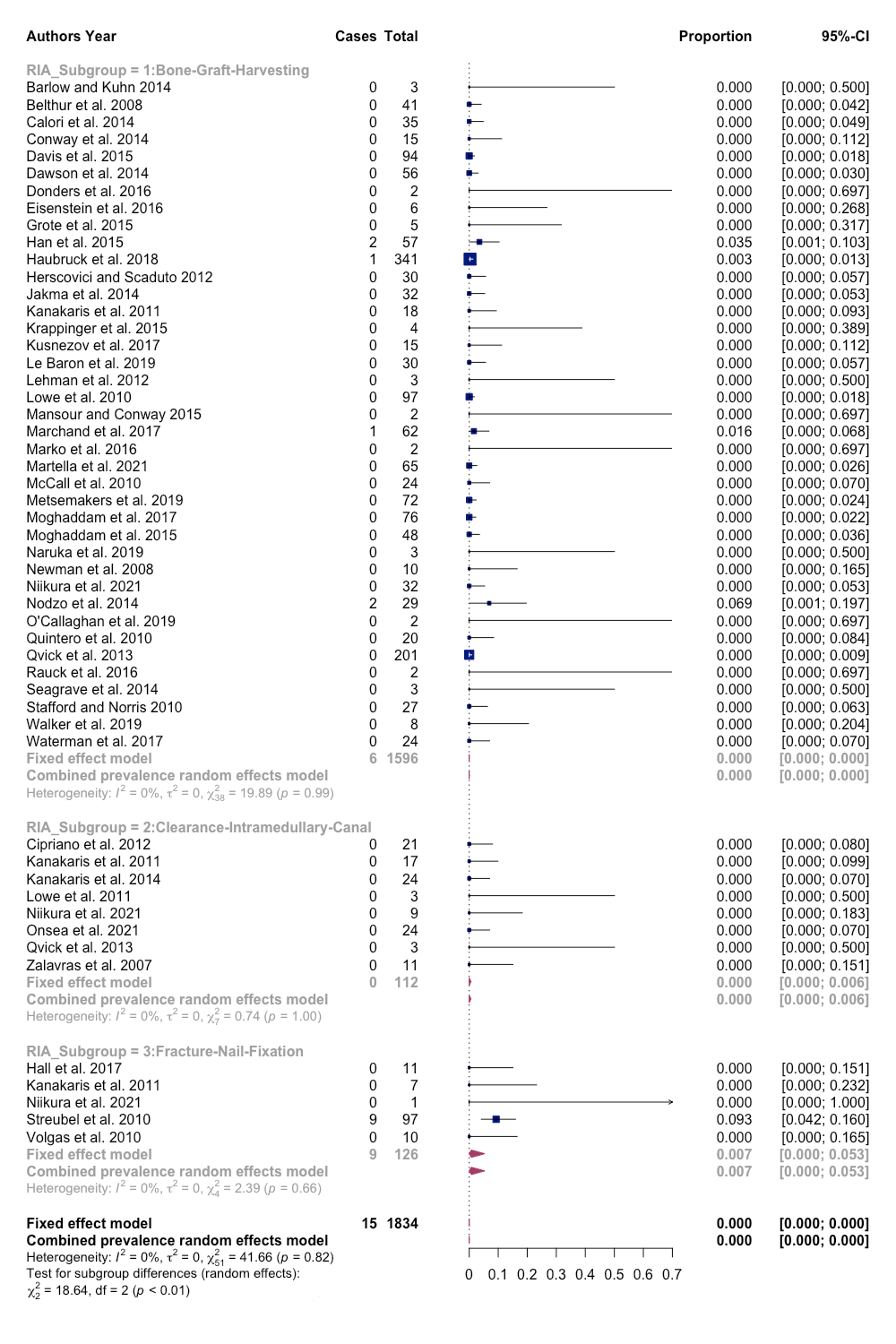
Wound or local infection

# References

1. Page MJ, McKenzie JE, Bossuyt PM, Boutron I, Hoffmann TC, Mulrow CD, Shamseer L, Tetzlaff JM, Akl EA, Brennan SE, Chou R, Glanville J, Grimshaw JM, Hróbjartsson A, Lalu MM, Li T, Loder EW, Mayo-Wilson E, McDonald S, McGuinness LA, Stewart LA, Thomas J, Tricco AC, Welch VA, Whiting P, Moher D (2021) The PRISMA 2020 statement: an updated guideline for reporting systematic reviews. BMJ 372:n71. DOI 10.1136/bmj.n71

2. Rethlefsen ML, Kirtley S, Waffenschmidt S, Ayala AP, Moher D, Page MJ, Koffel JB, Blunt H, Brigham T, Chang S, Clark J, Conway A, Couban R, de Kock S, Farrah K, Fehrmann P, Foster M, Fowler SA, Glanville J, Harris E, Hoffecker L, Isojarvi J, Kaunelis D, Ket H, Levay P, Lyon J, McGowan J, Murad MH, Nicholson J, Pannabecker V, Paynter R, Pinotti R, Ross-White A, Sampson M, Shields T, Stevens A, Sutton A, Weinfurter E, Wright K, Young S, Group P-S (2021) PRISMA-S: An extension to the PRISMA Statement for Reporting Literature Searches in Systematic Reviews. Systematic Reviews 10:39. DOI 10.1186/s13643-020-01542-z

3. Munn Z, Barker TH, Moola S, Tufanaru C, Stern C, McArthur A, Stephenson M, Aromataris E (2020) Methodological quality of case series studies: an introduction to the JBI critical appraisal tool. JBI Evidence Synthesis 18:2127-2133. DOI 10.11124/jbisrir-d-19-00099

4. Moola S, Munn Z, Tufanaru C, Aromataris E, Sears K, Sfetc R, Currie M, Lisy K, Qureshi R, Mattis P, Mu P-F (2020) Chapter 7: Systematic Reviews of Etiology and Risk. In: Aromataris E, Munn Z (eds) JBI Manual for Evidence Synthesis. JBI.

5. Tufanaru C, Munn Z, Aromataris E, Campbell J, Hopp L (2019) Chapter 3: Systematic Reviews of Effectiveness. In: Aromataris E, Munn Z (eds) JBI Manual for Evidence Synthesis. JBI.
